# Supplementary material for: Comparison and Functional Analysis of Odorant-Binding Proteins and Chemosensory Proteins in Two Closely Related Thrips Species, Frankliniella occidentalis and Frankliniella intonsa (Thysanoptera: Thripidae) Based on Antennal Transcriptome Analysis
Source: Int J Mol Sci. 2022 Nov 11;23(22):13900. doi: 10.3390/ijms232213900 (PMC9692942; doi:10.3390/ijms232213900)
Supplement: Supplementary file 1 [file ijms-23-13900-s001.zip › Supplementary Data Sheet S1 The OBP and CSP amino acid sequences used for phylogenetic trees.pdf]

>FoccOBP1
MDRPAALLCLAVLVALAATALCYPRPAPVVTPEQYEKSLKMLRNVCQPKTGMPADMLERMKGGEFV
QDEKAYCYTACFLQTMMVLKNNKVDSKMFKMQVKKMMLPDAAASVINAFTECEGTPAGAEPCETT
GLFINCVKKVDPTFNVPAWAASVAKSTMLHTPDMLLVPQDKNTPAFLYPQFLIKASICYKTMKTTAPK
NDKF
>FintOBP1
MDRPGALLSLLAVLLALSATALCHPRPRPAPLVTPEQYEKSLKMLRNVCVPKTGIPEDMLNRMKGGEF
VQDEKAYCYTACFLQTMMVLKNNKVDSKMFKLQVKKMMLPDDAARVIAAFTSCEGTPAGAEPCET
TGLFINCVKKVDPTFNVPAWTHHNAKPHRRLREREPRLFTWHH
>FoccOBP2
MSASTILSIVATVITSGRAAYNLDNPYYNSALFDQMDDFYMPKSENFAADSDPELRQKMSFSNEICC
GTYSDKNMMREDNVSRECYEEVFKSSDFLDSWNYFDTDAAKTVAQKVVCLQQCMWKKSGTMDD
RYDSLTGSLSDRYSEGSGSKEPNMNFPEVAVAKCLPSSNPDPASNWFGFGSSDCNRAYLDFSYCVW
EELHADCPSQLWDSDNKWCEETKEYLQARSQLTKS*
>FintOBP2
MDAPTLIGVVIATVVASASAATYNLDNPYFNRSLFDQLDDYYMPNSENFVAESDPELRSKMSFSNEM
CCGTYTDKNMIREDNMSKECYDQVFKSPDFLDSWNYFNTDSASGVAQSVVCLEQCLWKKRGTMD
DRYDSLTGRLSDSYSEGSGKKEPNMNFPEVAVAKCLPASNPDPAANWFGFGSSDCNRAYLDFSYCV
WEELHANCPARLWDFNNKWCEETKEYLLVRSQLTKKWSGLVKNVSWIVRNTPMMISCQ*
>FoccOBP3
MQGYTVLAAAALIAMASLAQADKKKELMPRYLKYTLECAKELNAEVGVCKEMMKEGNDNSADKYQ
PCKCVIACVAKKAKVMTDAGEANVEAFTAAVDEFEIKEWSDEWQRVKPLCEPEVKGKKDCVLGYDFF
TCGYDKSEIFRDVMKKFMGAMDKS*
>FintOBP3
MQSSTVLLVAALVAMASLAQADKKKELMPRYIKYALECAKELNAEVGVCKEMMKEGNDNSAEKYQP
CKCVIACVAKKAKVMTDGGDANVDAFTAAVDEFEIKEWSDEWQRVKPLCEPEVKGKKDCVLGYDFF
TCGYEKSEIFRDVIKKFMGAMDKS*
>FoccOBP4
MKTLLTLAAALLALCGLASAGVELSEDQKQLMKQLSSAYMAETGVDEATIDACKTGQFADDPKLKCY
LKCTYQQMTVMDDDGVVDADMLLTMLPEEIQPKAEPVVNACKEMRGADACDNAMMFNKCLYEK
APDYYMVV*
>FintOBP4
MKTVLPLVVVLLALCGMASAGVELSEDQKQLMKQLSTACMAETGVDEATIDACKSGQFADDPKLKC
YLKCTYQQMTVMDDDGAVDADMMLSMLPEGMQPKAEPILNACKDLRGTDACDNAMIFNKCLYD
KAPEYYMVV*
>FoccOBP5
MKVFVLLAVALVAYASADEAARERAKAIKKECKEQAGATDDDLKELKEKDELLSPVNKKLMACVFSKN
KIMQNNKFSKEGSMEVARELMSGRPESETKLKIAEQIADNCAKEIGNVETTGDEMAKLIFNCLKKQAK
EYRKAMATVPNQDISQSSW
>FintOBP5
MKVLLIALALAACAACAVADDAARERAKAIKKECKEQVGATDDDVKELKEKDQLLSPVNKKLMACVF
SKNKIMQNNKFSKEGSMQVARELMSGRPSSESKLKIAEQIADNCAQEIGDVETSGEEMAKLIFNCLKK
QAKESGFPSPHVW
>FoccOBP6

MKAVNVVVLVVAAVLAVASAEDNRDKLIPEYMAAAMDCVKDYEMDFTVCKEMMKDGVNLAEEKF
TPCKCVPACVAKKRKLMSEDGEYDVDAFTKAVNEFGYEPWSEEYKKVFPICKDSYKGKKNCEAAAAL
AVCAWKNSKMMRDTVGQYMGQMDGE*
>FintOBP6
MKAVNVVVLAAAAALVQPAAAEDNRDKLIPEYMAAAMDCVKDYEMDFTVCKEMMKEGVNLAEEK
YKPCKCVPACVAKKRKLMSEDGEYDVDAFGKAVFEFGYEPWSEEYKKVFPICKDSYKGQKNCDAAGA
LAVCAWKNSKMMRDTIGQYMGQMDGE*
>FoccOBP7
MHILPVAVLALAALAAVEAAAPQLVRGRCATPPAAPQRIEKVINECQEDIKLSILEEALSVLGESTGIISS
DAKTRQPGKSSARRQAFTEDESRIAGCLLHCVYKKVKALNSAGLPTGEGLVKLYAEGVQDASYFLATA
QAVNHCLGAAQQQGSLLPQAVKEPGQKCYLAYDLFECVSDQIMEYCGASP*
>ApisOBP1
MLNLKVMMFLCLSVIVVYCESDQVPINSSAAVESCLLETNMTRDEFEDMLTSPNARELTILKSHAHKC
MFGCVMRKNHIVNDGVVSKEVLSKYVLNFYGRPDYKRRLIIKDVEHIVDVCAKKVADESETDECELAA
TLVTCIVLEANKAGLVDDPARQI
>ApisOBP2
MKVSAATAVLVALVATVQSSDPCNISTCYKSGTTKPPMAVTPTHLPVQSSSTQTSHPQTTYAKDHVH
GSTTTKSGVNATVTTASGASVNGTEPPAVVKSSAGVTGNSTTPKPTMTEGHVALKQKLNTIAVKCKD
ELHAPQEIMALVSNTVVPQNEQQRCYLECVYKNLNLIKNNKFSVEDGKAMARIRFANQPEEHKKAVT
IIETCEKEAVIDPKTTEKCAAGRVIRNCFVKNGEKINFFPKA
>ApisOBP3
MISSTFYITLVFGIAMLISCGHGRFTTEQIDYYGKACNASEDDLVVVKSYKVPTTETGKCLMKCMITKLG
LLNDDGSYNKTGMEAGLKKYWSEWSTEKIESINNKCYEEALLVSKEVVATCNYSYTVMACLNKQLDL
DKST
>ApisOBP4
MRGNYSSMVFLLFAIGFQDIFCQKQEPSGKCRAPDKAPLNLEIIINTCQEEIKSALLQEALDILNDGNVE
QNTPNYSSRSKREAEEDLTNEERRVAGCLLQCVYKKVKAVDETGFPVVDGLMKLYNEGVQDRNYYIA
TLSAVRHCISIAQQLKQQQPSKSFDDGQTCDLAYEMFECVSEKIEENCGVENKSNN
>ApisOBP5
MSANSATIKCIAVAAILLQISVIFADAGHHRRGKELLDTEDSDFFRCKQASRKSCCGPENAMKRFGDK
DKVAADECYAQVAEKFATVTATTPKQDLFSAEAVKITKKKQFCLHECIGKKNNLLTEDGSLNKTFIADY
AMKSVFKEQWQKQVGQKALDKCLEETYIPWPAEDKENVCNPVYVQFQHCLWLQYESNCPANKIKI
TKKCEKTRNRYRMQKSTSN
>ApisOBP6
MQKVVFICIFAIICQTVFTAGYDRTWILRQKRGTNDDECRTLLPSSEKKLPSCCQMPNILPNLDSTWEK
CFETFKQFKDKPETKEYKEMAHGKEPPCLFQCIFMQSGLTTSDGKLNEDAITKKMSEGINNDEKWKSI
WQNSLNKCFDDVKQEDKKQILIMNTPAGRLMKCFLRDMYMSCPKNVWVESSECLNMKDLVQKCP
EMPPPVFKSPPKLI
>ApisOBP7
MVARKRMYNMLPTTVLFAIIAATVLKDCDAYLSEAAIKKTQQMLKTVCSKKHSVEEDVFTNIKKGIFPE
DNNNIKCYFACNFKTMQLINQKGVIDKKMFKDKMSMMAPPNVYKILLPVIEQCTGKDKGEELCQSSY
NVIKCAHSVDPKSLEFLPL
>ApisOBP8
MFALKVACLCLSVAVVFGENNQQNGPSDRSATIFQSCIAETKLSGDALKGFRSMSIPKTQAEKCMMG

CLMRKVNVINKGKFSVEEATKVAQKYYGTNEAMMKKAKDLIDVCAKKAQSTTEECALAGIVTTCIVEE
AQKAGLSGGPGSRSRRTVSPKFRRDAM
>ApisOBP9
MIIKKTLLLSVFVLFGCLFSINKADDADAKDKELMSKLFTVVFKCFKDADWGTCGEMITTKYDITQAKYK
QCTCHMACAGEELGMINASGQPEPAKFLEYVNKINNPDIKSQLQLIYDKCQNVKGSEKCDLAEQFAI
CAFKESPALKERVSTLMEMLVKMKPKSK
>ApisOBP10
MEHLRSTNVVFAIVMALLVVQSSTRPQPDEMEEIKRTLYNACAGKFPITEEIKNNAKNSIISDDPTFKCF
LKCCFDEMSMIDEDGIIDGDSLKAMAPDHIKPILEQVIPSCTKNVKQDGCEASFEFISCGIKLNPLIVALL
PL
>ApisOBP11
MSSSTFYITLLFGIAMLISCGYGIFTTEQIDYYGKACNASEDDLIVLKSYKVPSTETGKCLMKCMITKLGLL
NDDGSYNKTGMEAGLKKYWSEWATEKIETINEKCYEEALLVSAEIIATCNYSYTVMACLNKKLDLDKLT
>ApisOBP12
MEHLRSKNVVFAIVMALLIRPKTDGNDAIEEMKNTLYNICSTKYRITEELKNNVKKSIISDDPTFKCFLKC
CFDEMSLIDEDGIIDGDSMIQMTSDDDKLIAEQVIPNCIKTVKQDSCEAAFDFVSCVIKLNPSTAALLPL
>ApisOBP13
MSLLLNSGGCCGHSTTTVMTLSVAILLTVGQVHNLKCRTADHSRRHGEFIDIAEQCNNTTSGDRPAS
AVDGDYSTYASGGDNDGRWNGDGRRNNNNNREFSVNRQDFTGNRGSGGGYDRDQSSCSEARP
QQYSGNTGSRSKNHRQGSYPSADYDSGQSNSNYNRQSLPTRRYRRDDSNEKSKRQKAAVTGSNN
RLLGNNRFRNMTKTGGNQPGKGTYLDKMDACTIHCVFNQLEMLNSNSRPDKYSIVNIMTNQIKDVE
LKEFIQDSIDECFDTLELDSHNNKCEFSKNFAVCMENKAQRNCDDWDENLSANKINSAGLQDGTNQ
QDKRKGY
>ApisOBP14
MSSSTFYLTLLFGIAILISCGYGTFTTEQIDYYGRACKASEDDLVVVKSYKVPSTETGKCLMKCMITKLGL
LNDDGSYNKTGMEAGLKKYWSEWSTEKIEAINNKCYEEALLVPKEIVATCDYSYTVMACLNKQMDLD
KLT
>AmelOBP1
MASNTKQAFIYSLALLCLHAIFVNAAPDWVPPEVFDLVAEDKARCMSEHGTTQAQIDDVDKGNLVN
EPSITCYMYCLLEAFSLVDDEANVDEDIMLGLLPDQLQERAQSVMGKCLPTSGSDNCNKIYNLAKCV
QESAPDVWFVI
>AmelOBP2
MNTLVTVTCLLAALTVVRGIDQDTVVAKYMEYLMPDIMPCADELHISEDIATNIQAAKNGADMSQL
GCLKACVMKRIEMLKGTELYVEPVYKMIEVVHAGNADDIQLVKGIANECIENAKGETDECNIGNKYTD
CYIEKLFS
>AmelOBP3
MKTIVILLFTLCIVSYMMVRCDDITLCLKQENLNLDDIDSLLEDESERMLRKRGCIEACLFHRLALMND
NVFDVSKFDVYLNDTDMDMDLKDSIRKIIRQCVDNAKNEDKCLTAQKFSRCVIDYVKFHITQYMISN
ANSNTTSEEESSDNST
>AmelOBP4
MKITIVSLLCVIYCALVHADTVAILCSQKAGFDLSDLKSMYESNSEEQMKKLGCFEACVFQKLHFMDG
NTLNVEKLESGTRELTPDDFTEDVHEIIEQCVSKAADEDECMVARKYIDCALEKMKFLDDELEKIAGN
>AmelOBP5
MHVKSVLLLITIVTFVALKPVKSMSADQVEKLAKNMRKSCLQKIAITEELVDGMRRGEFPDDHDLQCY

TTCIMKLLRTFKNGNFDFDMIVKQLEITMPPEEVVIGKEIVAVCRNEEYTGDDCQKTYQYVQCHYKQN
PEKFFFP
>AmelOBP6
MKGLGVSLLVALLLVLLAIEDTMSKKMTIEEAKKTIKNLRKVCSKKNDTPKELLDGQFRGEFPQDERLM
CYMKCIMIATKAMKNDVILWDFFVKNARMILLEEYIPRVESVVETCKKEVTSTEGCEVAWQFGKCIYEN
DKELYLAP
>AmelOBP7
MKKFLVIFVYILSVAVIIRANGINEILKIMAVSMKDIRYCIIHMGLTFKDFIKMQELLQEEDISEGNIKKYLT
NYSCFITCALEKSHIIQNDEIQLDKLVEMANRKNISIDVKMLSECINANKSTDKCENGLNFIICFSKLLSD
MYEDTFEDTLKHKSYV
>AmelOBP8
MTIEELKKTIKNLRKVCSKKNDTPKELLDGQFRGEFPQDERLMCYMKCIMIATKAMKNDVILWDFFVK
NARMILLEEYIPRVESVVETCKKEVTSTEGCEVAWQFGKCIYENDKELYLAP
>AmelOBP9
MFKNYHFFFILVITLIFLYFGEADIKKDCRKESKVSWAALKKMKAGDMEQDDQNLKCYLKCFMTKHGIL
DKNAEVDVQKALRHLPRSMQDSTKKLFNKCKSIQNEDPCEKAYQLVKCYVEFHPEVLQTVPFL
>AmelOBP10
MKYSILLSLLITCLICSPSVHCGTRPSFVSDEMIATAASVVNACQTQTGVATVDIEAVRNGQWPETRQL
KCYMYCLWEQFGLVDDKRELSLNGMLTFFQRIPAYRAEVQKAISECKGIAKGDNCEYAYRFNKCYAEL
SPRTYYLF
>AmelOBP11
MKAAEIWLVSLYWYLILQIALVYGEISDIDEFREMTSKYRKKCIGETKTTIEDVEATEYGEFPEDEKLKCYF
NCVLEKFNVMDKKNGKIRYNLLKKVIPEAFKEIGVEMIDSCSNVDSSDKCEKSFMFMKCMYEVNPIAFI
AP
>AmelOBP12
MLYNNLTIVIILIMCGVQNLRARSVNIFQDIADCVDRSNMTFHELKKLRDSSEARIKLINEEENFRNYGC
FLACIWQQTGVMNGSELSTYNIAGIIEGQYHDDEDLKTFFHKIALTCEDDVHRKFLHVNDECDVALSF
KLCMLKAMRNYP
>AmelOBP13
MKTIIFIFAFCLVGILAVSEESINKLRKIESVCAEENGIDLKKADDVKKGIFDKNDEKLACYVDCMLKKVGF
VNADTTFNEEKFRERTTKLDSEQVNRLVNNCKDITESNSCKKSSKLLQCFIDNNLMKIFE
>AmelOBP14
MKTIVLIFGFCVCVGALTIEELKTRLHTEQSVCKTETGIDQQKANDVIEGNIDVEDKKVQLYCECILKNF
NILDKNNVFKPQGIKAVMELLIDENSVKQLVSDCSTISEENPHLKASKLVQCVSKYKTMKSVDFL
>AmelOBP15
MKTILIISAICICVGALSIKDFQNAIRMGQSICMAKTGINKQIINDVNDGKINIEDENVQLYIECAMKKFS
FVDKDGNFNEHVSREIAKIFLNENEINQLITECSAISDTNVHLKITKIFQCITKFKTINDILNS
>AmelOBP16
MKTFVIIFAICVCVGAMTHEELKTGIQTLQPICVGETGTSQKIIDEVYNGNVNVEDENVQSYVECMMK
KFNVVDENGNFNEKNTRDIVQAVLDDNETDQLIVECSPISDANVHIKISKIFQCFMKYKTITDILNS
>AmelOBP17
MKTIVIISAICVCVSAMTLDELKSGLHTVQSVCMKEIGTAQQIIDDINEGKINMDDENVLLFIECTMKKF
NVVDENANFNEKISSDIVRAVLNDNEADQLLAECSPISDPNALIKISKILECFFKYKTINQILNS
>AmelOBP18

MKTFVIISAICVCVGALTLEEFQIGLRAVVPICRIETSIDQQKEDDFRDGNIDVEDEKVQLFSECLIKKFNG
YDDGGNFNEVVIREIAEIFLDENGVNKLITECSAISDADLAVKSAKLLKCIGKYKTLKEMLSG
>AmelOBP19
MKTIVVIFAFCICVNAMTIEELKIQLRDVQEICKAESGIDQQTVDDINEVNFDVEDEKPQRYNECILKQF
NIVDESGNFKENIVQELTSIYLDENVIKKLVAECSVISDANIYIRFNKLVKCFGKYKTMKEVLNL
>AmelOBP20
MKTIVVIFAFCICVNAMTIEELKIQLHDVQEICKTESGIDQQTVDDINEVNFDVEDEKPQRYNECILKQF
NIVDESGNFKENIVQELTSIYLDENVIKKLVAECSVISDANIYIRFNKLVKCFGKYKTMKEVLNL
>AmelOBP21
MKTIVIISAICVCVGALTLEELQIGLRAVIPVCRIDSGIDEKKEDDFRNGIIDVENEKVQLFSECLIKKFNAY
DDGGNFNEVVVREIAEIYLDENEVNKLITECSAISDADIHLKSSKLIKCFAKYKTLKEIMNE
>DmelOBP18a
RVNAEGCLKHHNLTSAQVQAVAPSTPVADVPVAVKCYSRCLIQDYFGDDGKIDLQKVGKRGSQED
HVILSQCKQQFDGVTNLDTCDYPYLILQCYFKGKQSGTIAS
>DmelOBP19a
GVTEEQMWSAGKLMRDVCLPKYPKVSVEVADNIRNGDIPNSKDTNCYINCILEMMQAIKKGKFQLES
TLKQMDIMLPDSYKDEYRKGINLCKDSTVGLKNAPNCDPAHALLSCLKNNIKVFVFP
>DmelOBP19b
DEEEGSMTVDEVVELIEPFGDACTPKPSRENIVEMVLNKEDAKHETKCFRHCMLEQFELMPEDQLQY
NEDKTVDMINMMFPDREDDGRRIVKTCNEELKAEQDKCEAAHGIAMCMLREMRSSGFKIPEIKE
>DmelOBP19c
QTQAFDLAKLLPKTGTEPIWAVIDRNLPQVQELVTAARMECIQKLQLPRDQRPLGKVTNPSEKEKCLV
ECVLKKIKLMDADNKLNVGQVEKLTSLVTQDNKMAIAVSSSMAQACSRGISSKNPCEVAHLFNQCIS
RQLERNNVKLVW
>DmelOBP19d
KPHEEINRDHAAELANECKAETGATDEDVEQLMSHDLPERHEAKCLRACVMKKLQIMDESGKLNKE
HAIELVKVMSKHDAEKEDAPAEVVAKCEAIETPEDHCDAAFAYEECIYEQMKEHGLELEEH
>DmelOBP22a
TKEPEEVKIVSECAKENNVHRKKALDLLMSYRLKKKTHNVMCFINCIFERTNILQKVKEKVVKENHNCD
SIKDADKCAESFQKFQCLVKIEMKVRGIDRG
>DmelOBP28a
FDEKEALAKLMESAESCMPEVGATDADLQEMVKKQPASTYAGKCLRACVMKNIGILDANGKLDTEA
GHEKAKQYTGNDPAKLKIALEIGDTCAAITVPDDHCEAAEAYGTCFRGEAKKHGLL
>DmelOBP47a
RFAKININLGLTVADESPKTITEEMIRLCGDQTDISLRELNKLQREDFSDPSESVQCFTHCLYEQMGLM
HDGVFVERDLFGLLSDVSNTDYWPERQCHAIRGNNKCETAYRIHQCQQQLKQQQQNLLATKEVEV
TTTPAGSDETKP
>DmelOBP51a
LFESEANECAKKLGITPDYFENFPHSSRVKCFYHCQMEKLEIIANGVVTPFDLKVLNISPESYDKYGVKV
KPCLKLSHRDKCELGYLVFQCLKREFNL
>DmelOBP56a
SSLNLSDEQKDLAKQHREQCAEEVKLTEEEKAKVNAKDFNNPTENIKCFANCFFEKVGTLKDGELQES
VVLEKLGALIGEEKTKAALEKCRTIKGENKCDTASKLYDCFESFKPAPEAKA
>DmelOBP56b

QSAAELAAYKQIQQACIKELNIAASDANLLTTDKEVANPSESVKCYHSCVYKKLGLLGDDGKPNTDKI
VKLAQIRFSSLPVDKLKSLLTSCGTTKSAATCDFVYNYEKCVVKGISA
>DmelOBP56c
KAWVMFFIFYISFTRSLSVSLNMSMTRTLVPDPPNGTENKLSQEMLRACMRRTEISMSQLKLFHMSL
MNSDYNNDNDIAPTPVQSIGDVNNLGDLDFNGNSQMPYLDLKHNEPLQCFVSCLYETLDLDRYNV
LLEEAFKNQVQTIIQHEKAEIKECSDLQGKTRCEAAYKLHLCYNHLKTLEAEQRIREILERTEAENEGFGP
EGSDFIDGIQHSGEAMTTAKSE
>DmelOBP56d
ELQLSDEQKAVAHANGALCAQQEGITKDQAIALRNGNFDDSDPKVKCFANCFLEKIGFLINGEVQPD
VVLAKLGPLAGEDAVKAVQAKCDATKGADKCDTAYQLFECYYKNRAHI
>DmelOBP56e
SAVGLTDSQKAEAKQRAKACVKQEGITKEQAIALRSGNFADSDPKVKCFANCFLEQTGLVANGQIKP
DVVLAKLGPIAGEANVKEVQAKCDSTKGADKCDTSYLLYKCYYENHAQF
>DmelOBP56f
MKSSEKIKACLKRQLGYTITENTKFDAKEDSLQSKCFYHCLLEVKGVIANDAISSEQPRKVLEKKYGITDT
DELEKAEEKCHSIKASGKCELGYEILKCYQSITKH
>DmelOBP56g
QQANIDSSVSKELVTDCLKENGVTPQDLADLQSGKVKAEDAKDNVKCSSQCILVKSGFMDSTGKLLT
DKIKSYYANSNFKDVIEKDLDRCSAVKGANACDTAFKILSCFQAAN
>DmelOBP56h
NPDFRQIMQQCMETNQVTEADLKEFMASGMQSSAKENLKCYTKCLMEKQGHLTNGQFNAQAML
DTLKNVPQIKDKMDEISSGVNACKDIKGTNDCDTAFKVTMCLKEHKAIPGHH
>DmelOBP56i
GPIKDQCMAAAGITAQDVANRHETDDPGHSVKCFFRCFLENIGIIADNQIIPGAFDRVLGHIVTAEAV
ERMEATCNMIKSETSHDESCEFAWQISECYEGVRLSDVKKGQRTRNHRG
>DmelOBP57a
KESQPFDFFEGTYDDFIDCLRINNITIEEYEKFDDTDNLDNVLKENVELKHKCNIKCQLEREPTKWLNAR
GEVDLKSMKATSETAVSISKCMEKAPQETCAYVYKLVICAFKSGHSVIKFDSYEQIQEETAGLIAEQQA
DLFDYDTIDL
>DmelOBP57b
RHPFDIFHWNWQDFQECLQVNNITIGEYEKYARHETLDYLLNEKVDLRYKCNIKCQLERDSTKWLNA
QGRMDLDLMNTTDKASKSITKCMEKAPEELCAYSFRLVMCAFKAGHPVIDSE
>DmelOBP57c
QSLSLLEETNYVSDCLASNNISQAEFQELIDRNSSEEDDLENTDRRYKCFIHCLAEKGNLLDTNGYLDV
DKIDQIEPVSDELREILYDCKKIYDEEEDHCEYAFKMVTCLTESFEQSDEVTEAGKNTNKLNE
>DmelOBP57d
MPEKMSLRLVPHLACIIFILEIQFRIADSNDPCPHNQGIDEDIAESILGDWPANVDLTSVKRSHKCYVTC
ILQYYNIVTASGEIFLDKYYDTGVIDELAVAPKINRCRYEFRMETDYCSRIFAIFNCLRQEILTKS
>DmelOBP57e
NPCVSQNELSEYEAHQVMENWPVPPIDRAYKCFLTCVLLDLGLIDERGNVQIDKYMKSGVVDWQW
VAIELVTCRIEFSDERDLCELSYGIFNCFKDVKLAAEKYVSISNAK
>DmelOBP59a
LKCRSQEGLSEAELKRTVRNCMHRQDEDEDRGRGGQGRQGNGYEYGYGMDHDQEEQDRNPGNR
GGYGNRRQRGLRQSDGRNHTSNDGGQCVAQCFFEEMNMVDGNGMPDRRKVSYLLTKDLRDREL

RNFFTDTVQQCFRYLESNGRGRHHKCSAARELVKCMSEYAKAQCEDWEEHGNMLFN
>DmelOBP69a
VEINPTIIKQVRKLRMRCLNQTGASVDVIDKSVKNRILPTDPEIKCFLYCMFDMFGLIDSQNIMHLEALL
EVLPEEIHKTINGLVSSCGTQKGKDGCDTAYETVKCYIAVNGKFIWEEIIVLLG
>DmelOBP76a
MTMEQFLTSLDMIRSGCAPKFKLKTEDLDRLRVGDFNFPPSQDLMCYTKCVSLMAGTVNKKGEFNA
PKALAQLPHLVPPEMMEMSRKSVEACRDTHKQFKESCERVYQTAKCFSENADGQFMWP
>DmelOBP83b
QEPRRDGEWPPPAILKLGKHFHDICAPKTGVTDEAIKEFSDGQIHEDEALKCYMNCLFHEFEVVDDNG
DVHMEKVLNAIPGEKLRNIMMEASKGCIHPEGDTLCHKAWWFHQCWKKADPVHYFLV
>DmelOBP83g
KFLLKDHADAEKAFEECREDYYVPDDIYEKYLNYEFPAHRRTSCFVKCFLEKLELFSEKKGFDERAMIAQ
FTSKSSKDLSTVQHGLEKCIDHNEAESDVCTWANRVFSCWLPINRHVVRKVFA
>DmelOBP84a
LQDHAKDNGDIFIINYDSFDGDVDDISTTTSAPREADYVDFDEVNRNCNASFITSMTNVLQFNNTGD
LPDDKDKVTSMCYFHCFFEKSGLMTDYKLNTDLVRKYVWPATGDSVEACEAEGKDETNACMRGYAI
VKCVFTRALTDARNKPTV
>DmelOBP99a
ADYVVKNRHDMLAYRDECVKELAVPVDLVEKYQKWEYPNDAKTQCYIKCVFTKWGLFDVQSGFNV
ENIHQQLVGNHADHNEAFHASLAACVDKNEQGSNACEWAYRGATCLLKENLAQIQKSLAPKA
>DmelOBP99b
DHHHHHHDYVVKTHEDLTNYRTQCVEKVHASEELVEKYKKWQYPDDAVTHCYLECIFQKFGFYDTE
HGFDVHKIHIQLAGPGVEVHESDEVHQKIAHCAETHSKEGDSCSKAYHAGMCFMNSNLQLVQHSV
KV
>LmigOBP1
MWARLNDCAALLLLLAAAARAWDVNMKLTGRIMDAAKEVDHTCRSSTGVPRDMLHRYAEGQTVD
DDDFKCYLKCIMVEFNSLSDDGVFVLEEELENVPPEIKEEGHRVVHSCKHINHDEACETAYQIHQCYK
QSDPELYSLVVRAFDATIGD
>LmigOBP2
EMTPEFMEIVNKCKTEHEPTEDELKGMMALKVPESANGKCFMGCVLQEIGVVKDGKFDKEEAKKHA
ASKMTDKDELEKHMQLIEKCSQEVGGETDSCGIGPKLMECIKQFAPEFDIALPQQPSE
>LmigOBP3
DAEKMKEAVDKCKASENLDSLDGLKSSKSPSTEEEKCFIGCLMMDMKLLSSDGQYDAASTKDMINN
CEYLKDKPDEKSVALEVADDCAGKATGCSGHCECGPKAVGCLIKGMVDKGYEESFARIDKMLEKLDD
>LmigOBP4
MGTAVAAAVLLLVAVANAEDSLMEIVIREVKGCMDSEHLNSIADLRSYNEAKSPEEKCFLGCMLKKFK
ALDADGQYDAEGLKATIQHCPRMKAHPNIQQAALQVADECAGKVTGCSDYCTCAPLATRCLHEG
MKNKSFQTIFIALDEALDKMQS
>LmigOBP5
MALAARVFSATLMLTAVLFGDISTAGEVFTMSQLKAAVNECNDTYFLSQKNWDTVFTTGSLEDENDL
VAKCFFECVLEKTGAMDEKGNINSDITKAVFLASHEGTGTAVQGHDDLIDMCVPGRDETDICERGYA
LVKCVTVEELSRRQARK
>LmigOBP6
QLLLVSLALCLSAAVAEKQAPWCPTTASQGVQEDMGQCAEEIKDAILREYAKTVSSRRTRSAEMSEE

DRLLVGCMVSCLFRKGPHSRLQTGSKLLLAELGAMRLFSDGADDARYRNATATAVRRCSASSRSLLP
DDGGPRHECELGFFMFECVSDQITEYCQWQPE
>LmigOBP7
AAETKVMEGIKACMASEHLGSLGQLKANNEARTPEEKCFVGCVMKHLHVLNSEGQYDLALVKERAN
NCPELAKDPQKKADTLRVAEDCAAKVIGCSGYCECGVAAGECLAQGMEAKGHETIYDFLRKIVDKM
DV
>LmigOBP8
AVRLLLLLPPLLLLALSCVTAAPSITSTEMRMDMMVIQHCNETHPVALIDMNKALINKKIEPQNTVFKC
FVFCLLNKYEWMDDEGGFLIANMKHNLSDSHLDQLSIDFIVYKCSATGSSDKCERAYRFTECFWGEVT
KFPENSDEKYEDPDLFALYQ
>LmigOBP9
TDATATAAMDKASSAAVTACLLIAVAALHTQALSLEQLRQTSKIVRNMCLKKTGVDLALVEGIQEGQ
FPDNQDLKCYMKCCMGAMQVLRQGRYNVNAAKNQADKMLPPDLKGRFIDMLDACSDRGDGVD
DDCEMAYQLTKCSYETDKEIFLFP
>LmigOBP10
AISESMSRAEEAASKIDIPELFEECNETFTIPKVTLNYFFSHGRLQNENDYGSKCFVHCLTDRSGEIDSDG
NFDVDLIKVMTRRFPNETNIEGLNEMVETCVADRGETDFCERAYGLVSCLVKEKLARLGNSH
>LmigOBP11
VATMRSLLPVAVSAVLLVAPSKTLEPDFTKGISDVKACMASENLDSLDALRTNKEARTAEEKCFIGCIM
KFVEVLNSDGQYDVALFKDHINGCPEMAKDQQKKAALLEVAESCAGKASACSGHCECGVIVANCL
>LmigOBP12
AVILTAASTLWFAAAAFAAMVTTEIPTEDILQRVQVCNKTYPVSQEMLRSLASTGGLLSDESDVNTRC
YLECYERLGGTVNKDGKFNPEKAVTLLVSYYPKIAELGVDSVTEILKNCNSKSGTGQCMTSYLIRNCFIA
GLNAKSPHTSVFDTSSSHI
>LmigOBP13
CDPRHDTATMSALFTCCVAAWLLLAAALLQPTKGDDVWHNTDIPATMAECNATFRLGWRCWDNL
LSDGHVIDESKYQQKCWFYCLLDETGSMHADGAFDKDLLKTVLQGFPNGSSLAHLDETTYTCVAQR
NEVDLCERAYAVVKCIMTEELSRMHQSS
>LmigOBP14
MCLVVLLFGFVQSLSLLFVPDSCLLLFVVVMFYFPCKISQANGSFVMTMLCLEQTCKMMDQLHQTCV
GESGVSEGNIDAARKGNFIDDGNLKCYMKCIFVQMTCMSDDGVFDADTAIAMLPDNLKDVASKAL
NACKGEKGSDACDTAFKINQCLFKQAPKDYILV
>LmigOBP15
MFYFYAFTFCCLLWMLFHCSVNCVNVDIETIWRECNETFPASEEALISFGKNGTIPDENDSVARCFTD
CYGKKTTLLTSDGSLNWTTLDFLMRSYDMKPTAKETFGKCQKNTSNVECMKSYLSLRCVAETVESLTD
IR
>LmigOBP16
MNWGLWLPVTIALVLQLSISEALKCHTDEDTQNPDEFQEVAAMCMKNTSGSELNRNDRESKRNGN
NYHKTNFENTNDNWNSGGMGQTFPGYNSENEGYGFRGSGRCNANGDGYNGNRNNMNQNNM
NGMRQRPRNRNRRSGQQSETADVDLEDIEPCAVHCIFRQMGMLGDDAIPDRSAVAKVMLRGVKD
TEVKDFVQEAVEDCFDQVESDRKGSKCEFSKNVALCLRQKGRENCEDWGEQDGDQQSNQNKNG
NNNGNYSNNSNQYGNKKWN
>LmigOBP17
MKAFQICTLICAVVAHCMCDKEEAMKILRASVDKCSAGYGLSRETTQYIVRHNFTIKDENDENQRCFV

QCVGQEMGDFNSEGIFDVDHATETAEKWLEWNGRTKSNLREEMEECAKITGTGTCMTTYLITKCAM
KAGE
>SexiGOBP1
MLFLLRALPLLAAVLPLRADVNVMKDVTLGFGQALDKCRQESQLTEEKMEEFFHFWRDDFKFEHREL
GCAIQCMSRHYNLLTDSSRMHHDNTEQFIKSFPNGEVLARQMVELIHSCEKQYDHEDDHCWRILHV
ADCFKQGCVQRGIAPSMEMMMTEFIMEAEAR
>SexiGOBP2
MTSKCCLLLVAMATITAEVMGTAEVMSHVTAHFGKALEECREESGLSAEVLEEFQHFWREDFEVVHR
ELGCAIICMSNKFSLLQDDTRMHHVNMHDYVKGFPNGHVLSEKLVELIHNCEKQFDSMTDDCERVV
KVAACFKVDAKAAGIAPEVAMIEAVMEKY
>SexiPBP1
MAFCRSATMSVRVALVVAASMLVVVQASQDVMKNLAINFAKPLDDCKKEMDLPDSVTTDFYNFW
KEGYELTNRQTGCAILCLSSKLEILDQELNLHHGRAQEFAMKHGADETMAKQIVDMIHTCAQSTPDV
AADPCMKTLNVAKCFKLKIHELNWAPSMELIVGEVLAEV
>SexiPBP2
MAGAKWWFVCVVFALYLTSAALGSQELMMKMTKGFTKVVDDCKAELNAGEHIMQDMYNYWRED
YQLINRDLGCMILCMAKKLDLMEDQKMHHGKTEEFAKSHGADDEVAKKLVSIIHECEQQHAGIADD
CMRVLEISKCFRTKIHELKWAPNMEVIMEEVMTAV
>SexiPBP3
MGSHNVFVALVLLAVGMRVAEPSKDAMKYITSGFVKVLEECKQELNMNDHIIADLFHFWKLEYALLS
RDTGCVIICMSKKLDLLDANGRMHHGNAQEFAKRHGAGDDVASKIVQIIHDCEKKHERDDDECLRV
LEVAKCFRTGIHDLDWQPKVEVIVSEVLTEI
>SexiPBP4
NFWKEGYEFTNRQTGCAILCLSSKLELLDQEMKLHHGKAQEFAKKHGADDAMAKQLVDLIHGCSRS
TPDVTDDPCMKALNVAKCFKAKIHELNWAPSMDLVVGEVLAEV
>SexiOBP1
MKRSLCALVVVSAVLKLASGYYEEPSYTRDKRSPPREGKDLGIFHPYQDLIPRHCWSRPKNVDMYKCC
PIPQLYSDEVLDICGIEKIKEDEESPTKRSPTKLACKDGMCLMKESNLLDKDDRVDYEKLRAYLDQWAE
EHPNFTEAILEAKKQCAYPDGQEKDKAECEPDQIFVCLTAFIIWHCKFIDEPECKELQAHVDECRPYYQ
KPKEQDKKMAKKGKKFRS
>SexiOBP2
MITSSSILVLAALVQVLFAQQPDFQSGPPGPPGPHHKGPPPGAFPPGIPKSCWVPPREVNLFKCCPIP
PLYSDEVMQSCGFEKPSEDGPKPPKRHRRPDGTCKEGYCVMGNADLLQTNNSVDYEKFRSYLDNW
AASNPDFAEAIQIAKEDCAQDGGPAGPPVCEPDRLFFCLTSKIFWNCKLRDEDGCQALQQHMDECR
QYYTKPKEQIEGNAERR
>SexiOBP3
MLSMKTVIALVGLLLYEAGSVLGKNEDSVLRVISSSIGDTVLECQGEMDFKDEVIHNFMNFWNRTNSL
GTKDLGCALVCVFEKNAFLSPDGNTVLSNNVRQFLRASGADELMSLRTLDLFEMCKNEVKQVINRCD
NALELGKCFRYGIFQLHWNPEPHYWTRKEEPSKIPKQVTELQKAPRPLSRKPVPAWRRRTDLRRIVGY
VNRNCH
>SexiOBP4
MLKLCVFLALSFVACHGAPNKASGTFCGVTPNNMFKCLNNPRVLNLEAAAKCTSQVTECEKITCVFR
EQKWSKRGVIDKAKIRAHFEQYETEHPEWAQAVQHVKAFCLAPELRAQGVFLNCPAYDIMQCALAS
FIKHASPSVWSTEQNCDYPKAYAADCPVCPSDCYSAAIPIGSCNACYLQPRTV

>SexiOBP5
MFSHILHHALKLHMFYLFYLNKSSFFLSKLFLSYLTLSISFQSLSDREKSAIQKELTSVGLQCIQQHPLSLS
DIRSFRNKMIPDGKKPKCFVACLFKKIGVMDDMGMISPMKAQENAKKVFKDNEEYIKNVNEIMEKCS
SVNQQNTSDGNKGCDRAKLAFNCFTENADKVSYLF
>SexiOBP6
MKEGNRYSHERRITNDSGDQLMVINATDDDYSGYGSGNMGEKLLTSVPRPASSNNNINKNNTRRT
RRNEPLLNRPDSDQCLSQCVFANLQVVDSRGIPREAELWNKVQTSVTSQQSRSALHDQIRACFQEL
QSEAEDNGCSYSNKLERCLMLRFSDRKVDGKGNPKKSSTEQT
>SexiOBP7
MTRVLLAIGLTVITFALTQSANTKTSAMPKEAMTTTTMSDQDSSVDNNVDVDVIAVMNACNESYRIE
MAYLESLNESGSFIDENDKTPKCFIRCVFENVGIVSEDGMQLNPARAAVIFAGQRNGKPMDDIGDMT
ALCAADRQETCPCDRAYKFIRCLMSMEIERYEKS
>SexiOBP8
MSKFTFLVLCVVAVSLSKVYASDEDKAKLHEALKPLVEECMKEHEVSLDDLKAAKEAQSADGVKPCFL
ACVYKKAEVLNSKGEFDADHALDKLKEFVSDEDVLAKVAEVGNTCKAVNDKAVGDGDAGCERAALL
TACFLEHKAEVKPIRPLLFPWGHHHHH
>SexiOBP9
MARRQRGAMFTETLPLFVILVAVTHGGKDKPVFSDEIKEIIQTVHDECVAKTGVAEEDITNCENGIFKE
DAKLKCYMFCLLEEASLVDDDDTVDYDMLVSLIPDEYYERTTKMIFACKHLDTPDKDRCQRAFEVHKC
SYEKDPDLYFLF
>SexiOBP10
MSRFGVLSFVVLIFCMGNIYALSPEEELSIKEALHPFVVECAEEYGMTEEMFEEAKKKGSAEDIDPCFMS
CFLKKTGFFDDAGKFDAEKSISFAKEHITSESAIKFLVAGAGECVQINDEDVSDGDKGCDRAKLLFDCLT
DLKKKLSE
>SexiOBP11
MCKFSVVFLYSAVMAVNIWSASCLSEEDKAAIITAIAPLAQNCGSECGLDNDDFEKYKEDGSDMDPC
FKACLMTQMGVLDKEGKYDGKGLHKAMEEADYPGDKDDAQKFLDELDRCFDAKGDNSGSDEEAK
MKRADVLFQCMQDMKEN
>SexiOBP12
MFRFLVVACVAVVAVNGLSQELKEKFMERLETVGGECAAEVGANEDDIAELIAHKMPSRHEGECMIF
CFYKHFDMMHADGSLHAEGAIKMMEPLKADDPDLYEKLMTIGKACAEEVSSLDDKCKYATLLAQCG
VKKGKEMGLDESLFE
>SexiOBP13
MSKFTCFVLCVLAVSLAEVRSNALEKAAIRAALYPLIVDCAKEHSVTLEQLKAAKAAHSAQGINPCFQS
CVYKKTGIFNDNGEYDIANAKTKLQKFVTDEDEYARIAEVGKTCASVNDKSVSDGAAGCERAALLTAC
FLEHRAQIII
>SexiOBP14
MLGSLLFVFAFSVFSLSAEALLIDDLKQKYVDSILQCSQQYPLDRADAELLQNKVMPDKESTKCLFACV
YKVTGVMSDQGELSVEGVNALSQKYLADDPEKLKKSEEFTEACRTVNDAPVSDGARGCDRAALIFKC
TIEKSPDFSFV
>SexiOBP15
MSKFTCLLLCVVAVSLSKVHATEEEKEAIRAAVRPIMEACGKEHGVTLDDLKAAKAAHSADGIKPCFQ
SCVYKKAGIFNDNGEYDIANAKTKLQKFVTNDEEYARIAAVGKTCASVNDKPVTDGAAGCDRAALLT
ACFLEHRAQIII

>SexiOBP16
MSKFTCLVLCIVAASLSQAYASEEEKAAFREAIKPIVEECSKEHGVSHDELKSAKDNQNADNIKPCFLG
CVYKKAEVFNSKGEYDVDKALEKLKKFVSNDEAYAKFAEVGKKCASVNDKAVSDGDAGCERGALLTA
CFLEHKAEVPL
>SexiOBP17
MSVVRYSSFVMALFCLVSVNAMSGDEEAGVRDALRPYVQECADEYGITEEQFEEAKKKASADDIDPC
FMSCFLKKAEFFDAQGKFDVDSTMAFAKEHLSSEPAMKFVEAVGDECVKINDEDVSDGDKGCDRAK
LLFDCIAETKKKMD
>SexiOBP18
MSKFTCLVLCVVAVSLSGVHATAEEKAAFIEAVKPYIQECSKEHGVTPEDIKSAKEAGNADGINACFLR
CVYNKAGVINDKGEYDADKALEKLKKFVSNEDDYAKFAEIGKKCASVNEKSVSDGEAGCERAALLTSC
FLEHKSEVHA
>SexiOBP19
MTSSSVVWCAVICISTVFAWYDEPYNKKGFDECIEKFHVQPREKGSFQKPDISSVDPCFWACGFKILG
FLDSEGQYDLETTHSHYKKENLSYLGEKQEKVEEIIEQCDAALEKITGTDPKAECDRGFQLAKCYVEDM
RKLLFEDSRK
>SexiOBP20
KFLFLVACALVAVNAVSEQLKNEFIEKMTNIGGQCAKEVGANEEDIAELLAHKAPSRHEGECMIFCFH
KHLGLMNEDGTFSKEGGLKALEPVKADDPKLYEKLVSIGKMCQEEVAKDDDKCKYATQLTVCGVKKG
KEMGLDASMI
>SexiOBP21
MSYINYFLFSVVLFVICNNSFVYSMTREQIKNSGKLIKKTCSAKNDLTEDEVKDVDKGKFIEKKDFMCYI
ACVYKMGQSVKGSTINHDMMLRQVDMMFPNDMKAPVKAAIEHCRPVAKNYKDLCEASYWTAKCI
YDFDPANFMFP
>SexiOBP22
MKTYTFRLFCYIFCISLFLGQSYGMTRQQLKNSGKLMKKSCMPKNDVTEEEVGDIEKGKFIENRNVMC
YIACVYTMSQVVKNNKLSYEAVIKQVDVMFPAEMRDAVKAAASYCKDTTKKYKDLCESSYWTAKCM
YDYDAENFVFP
>SexiOBP23
MMDRKRLCFLIIAMYLAQGSDAMSRQQLKNSGKMLKKNCMSKNQVTEDQIGSIEKGKFVEDKKVM
CYIACIFEMTNVIKNNKLNYDSSIKQIDLMYPPELKESAKAAAEKCKDVQKKYKDICEASYWTAKCLYD
FKPEDFIFA
>SexiOBP24
MQINQLLGLLVIATCVGISHGMSRAQVKKTMSLVKNQCMPKNSVTEDQVGKIEEGVFLEDRNVMCY
VACIYKNLQVVKNDKLDMGLITKQIDALYPPELKEPVKKAVSLCIHSQDNYNDLCEKVFHASKCLYEK
DPASFIFP
>SexiOBP25
PTFTYLLNTELQARTDQEIKAWFFREGMDCNNEHPLSPKEMLELKENKIPDTNSAKCFVACVFKKTGM
LDSKGMFDAESSIAMTQKDFVDDPKKLESSKKLLEACKKVNDEAVSDGEKGCDRSVLLHKCFVETAP
QLGIKLP
>SexiOBP26
MKVFVLLVLSAYILIENEAAMTDAQLKAALKLLRNVCQPKNKATNEQIEAMHKGDWNQDKNGMCY
MHCVLNMYKLIKKDNTLDYEVGMSTIEAQAPDSIKATAIHSLNSCKDAAKTTSDKCIAAFEIAHCLYLD
NPPAYFLP

>SexiOBP27
MLLTKIIKFLILVATCEAMTMKQIRNTGKMMRKTCQPKNNVEDEKIDPIAEGVFIDEKEVKCYMACIMK
MANTIKNGKLNYDAAIKQADLLLPDDIKEPAKEAITACKKVADAHKDICDASFHITKCIYNHNPGIFYFP
>SexiOBP28
MKTFVVLAACVMLAQASGLTDEQKEKLKKHRSECLTETKVDEQLVNKLKGGDYKTESEPLKKYALCM
MMKSELMTKDGKFKKDVALAKVPNAADKPTVEKLIDACLANKGNTPHQTAWNYVKCYHEKDPKHA
IFL
>SexiOBP29
MFFVQAISYSTILVLFMVTSHVRTDSSEEEEKLMVKCMEEASVTKEEVKVFRTDKISDKILCFMKCRFESE
GMFDENGVIIKEMLQEGYDDFGWNDEQKIKADECIDNMKPAKECGDLADFFSCLPVINYGELIK
>SexiOBP30
MNYAIFFFLVAILSNASGMDDDMQELINNLHNTCVGEVGVDEALITKAQNGDFAEDEKLMCYSKCLL
DQMAIVDENGIVDPEAAVAVLPADMQAEAGPAVRKCSKLRGSSPCSNVFEVMKCWYTESPATYFLP
>SexiOBP31
MKFLVVAACVLLTVQALTDEQREKLKEHSTACAKSTGVDPEAIANAKKGTFSDDEKFKDYLFCVSKKIG
FQNEAGEIQKDVVKQKATVALKDEKLVDEIIKKCAVVKDTPQNTAFEVAKCYYENNAKHSSLV
>SexiOBP32
MKSLVVFCIVLVVGVCANEKGNKLASECIKETGVKNELLEEAKKGIISEDPAFKAFTYCFFKKIGIVGEDGI
LNRDVAIAKLPSGVDKSEAEKLLDSCKSKTGKDAVDTVFEIFKCYQQGTKSHIMFAS
>SexiOBP33
FTCIILCVVAASLTKVSHAVTEEEKEAFREAMAPIIAECSEEHGVSEADIKAAKEAASADGIKPCFLGCVM
KKIEVLDSKGLYDAETGLGKLKKFVKDEDEFAKFEDIAKKCLKVNDESVSDGEAGCD
>SexiOBP34
IAETKKKCLSDEDKKILRDGLDSIAHDCLHGCGIDEKELDNLDANDSIECFKKCFMTDAGFLDLNGKYN
KDVLSESLSKVTGNQDNAERILNELDRCFTENGDNSEANEEAFMKRIDILFACMREIRE
>SexiOBP35
SLKVLSVFVALIVALHADDDHEKHFAMFKECAEENGLKMDGFKRGERPVGPPSNEMMCTVKCTMEK
EGILSGGKILIEEFKKDPKLTKHVPADKMDAALECLKGVEVSDCSDMKKVMDCTHDMKF
>SexiOBP36
EHTMPAQRRRDKREVPFTHDEKRIAGCLLQCVYRKVKAVDGYGFPTLEGLVGLYSDGVNERGYFMA
VLEASRECLMKNHDKFSRTVPMDNGRNCDVSFDIFECISDRIGEYCGTSGL
>SexiOBP37
QKAQIHAHFESVGMECNKGSNMITADDIASLRAKKIPAGPNAPCFLACLLKHIGIMDDSGLLQKETAL
EMAKSVFQDPEELKQIEDYLHSCSGVNAESVSDGAAGCERAMLAYKCMT
>SexiOBP38
MAELARMVRENCAGETGVDVALVEQVNAGAELMPDDKLKCYIKCTMETAGMMADGEVDIEAVLAL
LPPSLAEHNAPALKACGTQRGADHCDTAFRTQQCWQNANRADYFLI
>SexiOBP39
DKIAIMSAMKPIVDECAKKHGVTLEALLAAKASGKIDGIEPCFYSCVYKKTEFLNSKGEYDVDNSLVKLK
KYISSDDDYAKFSQIGKDCA
>TcasOBP1
MILKASIFLILAVATFGAILEDSELMKVVENCVKKTNANESEFSSPNFLETTPSQPALCTAKCLLESLEIVN
SEGNINMETLKEYAQPFESPAREAVATCGEEIKSVTTCDDMEKYRKCVEPLIKNS
>TcasOBP2

MNPITSVILTFLFVFSFGEKESEEALQQIFTELDGPAAELRDQCLEKNSMKVTDLKTYNTSNDIPEKELCF
YKCFYEGVEFIDANGNLNVNNMKEIPAISELGDEVLNEITACVEKIGKIRCCGDLRKIEQCYQNITM
>TcasOBP3
MWSFVTLLFSFLVLASAQKKGKYWTTISECLTEHSMGVEDMKKFDLPAEKMSEEMLCFNKCFYDKLLI
TDENGEINTDNLMSIPLVNAIDASKHDDLVTCLKKVGKIEECDGVKKIEQCFVEFI
>TcasOBP4
MCRLFVVLSLFVASQALDVEKIRNELMADKNFVELRNKCLDKLGLKEEDLRDLKFDGDVSEDLMCFGK
CIQEEDGLLDSEGNLNEEKLEKKIETMPFLSRVSDDTKNNIMECLKEIGKIETCQDFGKQRDCIHKYV
>TcasOBP5
MAKKQLVLFFLAFIFLQSSWAYFFMSQKFAEVREECLSENSMTMDELHEGWKMENLPESHLCFLKCLL
EKREVIDENGVPQKEKIDEILTVKQLSDEKREEISTCITNVEKIENCETMSEIMRCFPKKRRD
>TcasOBP6
MSPLLLIFISCLFPRVFGISEEMQELANTLHATCVDETGVSEDAIESARKGNFAPDDKLKCYMKCIMEQ
MACIDDEGIIDVEATIAVLPEEYQAKAEPIVRKCGTKIGANACDNAFLTNKCWYEEDPEDYFLV
>TcasOBP7
MYKTRVIYVLFALCLVEIFAIEMDDDMKELINNLHNTCTGETGATDDQIENARKGNFAEDDSFKCYFK
CVFDQMGCMTDDGKVDSEAVIAVMPPELADKIASTVRGCTEVGANPCETAWLANKCYQKSNPDM
YFVP
>TcasOBP8
MIRYYIVLLLYFFAPPVLGISEEMQELVNQLHSTCVAETGVSEDLINKVNSDKVMIDDEKLKCYIKCLLTE
TGCISDDGVVDVEATIALLPEDMKAKTTPVIRSCGAKMGANPCESAWLTHKCYLETSPADYVLI
>TcasOBP9
MKAILLLLVATLSFYHVYCAMSEAQMKAALKLVRNVCQPKTKATNEQIEAMHTGNWDLDKNGKCY
MWCILNMYKLIGKDNSFDWEAGIATLKAQAPESVRDPAIASVNNCKDAVKTTSDKCEAAYEIAHCM
YLDNPEKYFLP
>TcasOBP10
MKTVAVLLFLALAACTKQEDDDRQETIRQYRDDCIAETKVDPALIDRADNGDFTDDAKLQCFSKCFY
QKAGFVSETGDLLFDVIKDKIPKEANREKALAIIDKCKELKGADSCETVYLVHKCYFLHSYGTDKKTE
>TcasOBP11
MSFLILLICVIPAIFCRSFSHDELDTDLSFIKTCNRTSPISMRTMNEVLINKKLGHGESSAFKCFLHCLFMK
YGWMDSDGGFLLHDIKQTLEESDVEIASLEFILYKCTATESNNRCERAFVFTQCFWDKMAEQQPSED
QFFYNIEDKK
>TcasOBP12
MKLLITLATLVVATYAIDKEFVQELRQKLRSHVEACAKEVNAGPDDVSAIFAHKLPATHEGKCIFFCMH
KLYNAQNEDGSLNMAGALANLELIKDMDPDVYTKVSTSFKNCESAPFDSDPCLYAANLVTCIVKEGR
AVGLDEVLVE
>TcasOBP13
MKFLLVFLSVAILCTFAMDESFLQQTRDRVKAIVKECVTEEKATDSDFDDIMALKIPTSHEGKCVFFCSH
KKFNMQHPDGSINKEGALDTFEVVKDVDAEFHDKVITVYNHCLSTPVDPDPCVYSVNLFQCFMKEA
KAAGIHELIIK
>TcasOBP14
MNSVLFLLVCALVACSGELDKEFLMQFLQKIKKVSEDCIAETQATKNDIKTLLEHKIPDSHEGKCMIFCF
HKHFQIQNEDGSLNKVAAISLLEPIKDHSQDIYDKVVKIFNTCFDSAERDDDSCIYASNLAECAIRESKS
LGLDDLLVIE

>TcasOBP15
MNCFVIFALSLSATVFGQSLSEDEMRENARKLMTSCKDKVGASDADVEALKMHQMPESREGFCMLE
CVFDSAKIMQDGKFSKSGMIEGFKPLIGDDKAKLESLEKLSATCESELGDGEDKCETAKRLVECVIKNGK
THGFEVPPPRE
>TcasOBP16
MQLLVVVLAVCVLGANAGLDPKFLEKLTQEVQAVGTSCGEKEHATADDMIEIMEEKFPPTSHEAKCV
VACFYKHYKMMKEDGTFDKDAAVKAFDEIKAQDAEIHAKILKVIDACDAKKQMSDDHCVSAASMA
GCVKTEAIANGLTKEAFMAS
>TcasOBP17
MKSTWFFLLLACSLTCALDQEFVDEFLEKMQEFGAQCAEETDATSDDIAELIARKLPPSTHEGKCMIFC
MQKKFNMMKENGGIDRAGAIAALKPLQKADPELHQKVLKIFVTCGMRVKPSPDPCDTATELALCGK
KEAEAIGLEDALLT
>TcasOBP18
MKLFILAGILFTGVCAVDQEFVEKFLQKMEKIGEECAEETHATSDDIADLIEQRDPKTHEGKCLIFCYHK
KFNTMKEDGSLDKVGSVLALEEVRDADFELYKNILTIFVTCGDKAKIYDDPCETATALTMCGRDEAKAL
GLQDAIFG
>TcasOBP19
MKYFVVFASLFLATNALSQDFIDKFVAKVKSIGETCVPETNASKDDISSLLAHKMPDSHEGKCLIFCFHK
QFQIQNDDGSINREGAIKALEPLKADDAELYEKVISIFKKCESTPVDGDSCLYAASLAECAVKEGRAMG
LDNLIVLEIE
>TcasOBP20
MATRFCFGLLILFVGTVLVFAENEHEILEVRALCMNETGVSEETARNYKPAEDPASEEILCMVKCIFEKIG
CLKDDGSFCVDTMKKKNYIMDVINEENEEKIYECLRGVGKITNCRDMAAVEECFVKNDSK
>TcasOBP21
MLRLVSLLCLFLLVQGENLDMFDPAGLQACMKKLSVGETELAKALEDKSKDPPEKIMCLFKCALEDSG
FLQDGVVDKSKWPMPECVQDVVKITNCNDMVALKHCFD
>TcasOBP22
MKPIFAIITLTLCTTVHALDCGIHINKNDALKATINKCLISNKTLEDLWDMAPMSSESDSSSEEVPPVDG
KMLQNFRIKRASVRLTNTETNETTPEPKAVSSEAQATENCIIQCIFDNLQMTDSTGYPVHTKILDGLLK
NTTNRELRDFLQDTTDECFQVMDKEDTMDPCSYSNKLVTCLAEKGRSNCADWPVGELPFKP
>TcasOBP23
MKYFPHLCLCLIFFELSEAAMSEAQLKAAVKLVRNMCQPKSKATNEDIEKMHHGDWNIDRTAMCYM
HCALNSNKLITKENVFNRDYAITLAEKNLPTALKTASIEAANLCKDSAKTLDDKCVAAYEISKCLYESNP
EKYFLP
>TcasOBP24
MSRMLPAALFVVLATLTFATAEIVVPDDLKDYINELHDHCLKEMGLTEGDHKNYNIHVKDPKMMCY
MKCLMTTSKWMNMDESIQYDFILSSVHPAVKNILLPALDKCRDIPKGTMECEKAYNFNMCLFNADP
ENWFFI
>TcasOBP25
MPLKNLIILIVCPLFVFAKVDIPPDLQAEIDGYYDICYKQIGLTKDDLKAYKIGDRDPKIMCFMKCVFVEA
KWMDENENLQYDYIKNTIHHSIRHITLPELENCGKKAEGDKCEKSFSFFNCMNKAEPEDWVLIQ
>TcasOBP26
MMHLKNFVVLVVCPLFVFAKVEIPPDLEAEIDEYFEQCFEPNGVTMDDIKAYKMGDKDPKIMCFMRC
LFVSGKWMDENENMQYDYIKETIHHAIRHITIPELENCGKEAQTGDKCEKSFNFFMCMNRAEPEDWI

LDYKS
>ApisCSP1
MNLLAIFCYITMMCDSQFRRLEQMTAMPQVKQPATIATRIGQATIAPRFGQPTIAPRFGQATVAPQV
GQAAVTPQIGQAAIGSRIGQSFQSVNGSVTPTTDGRKTTRETASYPTRYDFIDIEAVMNNDRIIKILFN
CVMNQGPCTREGLELKRIVPDAIQTECAKCNERQRKQAGKVLAHLLQYKPEYWNMLVKKFDPNNIY
LRKYMADNDDDEKLSLQKLTNNTTK
>ApisCSP2
MAHLNLFVVLVASLVCFTLAEEKYTTKFDNFDVEKVLNNDRILTSYIKCLLDQGNCTNEGRELKRVLPD
ALKTDCSKCTDVQKDRSERVIKFLIKNRSAEFDKLTAKYDPSGEYKKKIEKFDAERAAAAKH
>ApisCSP3
MVHLNLFVVLVASLVCFTLAEEKYTTKFDNFDVEKVLNNDRILTSYIECLLDQGNCTNEGRELKRVLPD
ALKTDCSKCTDVQKDRSERVIKFLIKNRSAEFDKLTAKYDPSGEYKKKLEKFSA
>ApisCSP4
MDSRIALVCVVLAVFAVDQTVGAPQKDAASGPVYTTKYDNIDIDQILASKRLVNNYVQCLLDKKPCT
PEGAELRKILPDALKTQCSKCNPGQKNAALKVVDRLQKDYDKEWKLLLDKWDPKREQFQKFQQFLV
EEKKKGVVKF
>ApisCSP5
MNCKILIALCCVAVYAAQANPAGVATATAADEEIKDLPAYMKRFEKLNVEQVLNNDRVLASHLKCFL
NEGPCVQQSRDLKRVIPVIANNSCNGCTERQITTIKKSLNFLRTKKPVEWARLVKIYDPSGVKLNKFLD
A
>ApisCSP6
MNKLFLAVAFCIVTMMTVVQTAPAKYTTKYDNVNIDDILNNDRLVNSYFKCLMETGKCTPEGEEIKR
WLPEAIENKCEDCSEKQKLGSEKIIKFLIEKKNDMWKQLEEKYDSKGLYRQRYSEDAKKLDIHI
>ApisCSP7
MARSSSSVTMKVFVIAVCVCAALARPEEAKMENKPAVVKSETLAAPLPTTIVKRATPYVVSTQQDSSL
PNVSEDVLDKALSDRRFVQRQLKCATGEGPCDPIGRKIKAHAPLVMRGMCVKCSQSEIKQIQRVMS
HIQKNYPKEYTKMLKQYQSGF
>ApisCSP8
MTNNNMNCPRSRPEIFSLLTVTAIAAVLVHQPTTVYCADGGTYPQQQLQQQQQQQQQQQQQ
QFTAPSGYYVSTYDHIDVGRLLRNQKVVSGYVKCFVNEGPCTPDGKLVKAYLLPEIIRTVCGKCTPRQK
EMARMVLRHIYTYRRADFDKIMQIYDTDGKKNEIINFMNQK
>ApisCSP9
MSSFCLNSVILMTVITVVVARVAFAESTTSNDRPGSDIRLVKKDVDYNEDDADDREEGFFFRISHFFGF
TSYDDDKPDFITTFDLIRLLDEKYAMKQFYCVINEEPCDAVGLRLKATIPEEINRDCERCTATETSNIRRIL
NYVKKHYPKFWERVEPIYRNNTTA
>ApisCSP10
MVSKRFISVFMFMAVVGVSFSVPEDDDATKVVNKEVDHHSVIQEEIKKFLSMMEKINIDQILNNDRL
MSNNVKCFLNEGSCTAQLREMKKMLPVLIKDSCSSCTKEQRNMIKKAMDAIKARRPNEYERVTKFFD
PEKKYEKKLSEKLNES
>AmelCSP1
MRHNYIVILILSLLTWTYAEELYSDKYDYVNIDEILANDRLRNQYYDCFIDAGSCLTPDSVFFKSHITEAF
QTQCKKCTEIQKQNLDKLAEWFTTNEPEKWNHFVEIMIKKKDEGA
>AmelCSP2
MASAIKALLIVCALFIYTVTAETEEGQSGRSRVSDEQLNMALSDQRYLRRQLKCALGEAPCDPVGRRL

KSLAPLVLRGACPQCSPEETRQIKKVLSHIQRTYPKEWSKIVQQYAGVS
>AmelCSP3
MKVSIICLVLMAAIVLVAARPDESYTSKFDNINVDEILHSDRLLNNYFKCLMDEGRCTAEGNELKRVLP
DALATDCKKCTDKQREVIKKVIKFLVENKPELWDSLANKYDPDKKYRVKFEEEAKKLGINV
>AmelCSP4
MKTILIALVPVCFLLGEVFSEDKYTTKYDNVDIDVVLNTERLLNAYVNCLLDQGPCTPDAAELKRNLPD
ALENECSPCSEKQKKIADKVVQFLIDNKPEIWVLLEAKYDPTGAYKQHYLQNRVKEESY
>AmelCSP5
MKIKILLFFTILALINVKAQDDISKFLKDRPYVQKQLHCILDRGHCDVIGKKIKELLPEVLNNHCNRCTSR
QIGIANTLIPFMQQNYPYEWQLILRRYKIMKYY
>AmelCSP6
MKIYILLFVLVTITCVIAEDYTTKYDDMDIDRILQNGRILTNYIKCMLDEGPCTNEGRELKKILPDALSTGC
NKCNEKQKHTANKVVNYLKTKRPKDWERLSAKYDSTGEYKKRYEHGLQFAKNN
>DmelCSPA75a
MKWLVIIVLLQLLEKIKCEQSYEVTNERLEPFEGDSQTLVLFDGLKTIGRERALNGSFKFLGEMNNDDFK
VSVELYSSPNGDGEFKRMVMDVPQTSICECFKKFYVQFVQPSLKTGETTNFPVVDDDFCPVPEGEFYV
KNVILNTQDWPSQVPRGIVKAIITFFSGGKNVGGLIVEVKIEDRQS
>DmelCSPB38c
MFATLLLILILGSTDILATDYILLVEDPDIYTPCTDGPPGSVGLNEAFDVSEMQVEMDEEGIHVSGNITTR
WSLPPTYRISARMSVLHFNRGNWEPTVFNTLTPDFCDAMFNPNLFWYKYWFKNFENREEIQEKCLAT
QGTVLVYNPFVVVPRLNNVLGPTLKGRYKVVFLFEAFNEQDERQPSSVCFEITGDAEKIKN
>DmelCSPA87a
MGSSLCPVSYLAVLAIIVLTSNITVQAKRTFRIQKLEKVTEDTSYLRSRLRIAESEENELKVSGYLDLNQRL
DNDWTVVLKVSRSPDSDGDYEKVLTFEMQLCDFMKSYYKDIFYERIKEYSNAPHPSSCPLPKERYVLE
DYPFNVKLLKKLMSPGFYRIKYTLKNEETKILSYVLDLELEEN
>DmelCSPB93a
MGCETSVLCTLLIFFQIIGRSFEMSHDFVPIKDDLLSKCEDKPEMGYLDAFVDLSNFSRKRGPGGVNISG
NITTIWDVDPSDVVEIDVSILKFEGDKWIPTIIKGNVKDFCKSFYDKNTLYYSYSTKHVVNKKEAKEKCIT
TPGAILLWEPYLLKISFSYAVPLNVGRHKAVLIFTAIDKAGVKRDRDICMEIVGDIVNA
>DmelCSPA98a
MDAKIIILALVSLFYKANGAFYELSVADEEIFSSCPNPEPGTLDIHGLFDFSEFSTSLEADGLTVSGNQTLV
WDIQRGDRVQLFIKLFYFDRGTWTSTAFSILSQDFCKTMYDKSNVLYEPWTGHVMNDVKDQCINAP
GTKLILDTYFLSLSASVTVPLREGRYKTTIKFRAFDSKGTERPTSICCEVIGDVFKIRN
>DmelCSPB42a
MKATFTILVLQVVICLAGATEYQLTLDKDGLLAPCENQPGNPSGFEAMVDTSSLKVHNLGSKVRIEGE
QKVVWKDVQPGDTLKVFGQVYRLDKGTWQKTMFTASSNNFCKNMFDKNQYWYNFWTKYISNSD
EIKEKCLTTPGAVLKYKDYELDLKTSLNVPNLDGRYKLVVQIEAFDKRNVRRPVPICIEFRGTAGQV
>DmelCSPB93b
MDRQQVTLLNILISLTLQIISPSFALNHFFIPEREELFSECLDKPGFSYVNELADLSRFNRKKDADGAMNI
SGNITMLWDVEPSNRVAVEVNIEKFEGGTWNPTLFKGGDKDFCKNFYDKNTIYYPFSTKHVINKQQV
KDKCITTPGTVLVLEPFILKILINFAVPLSPGIHKAVIIFSAFDKSGVKRPRDTCIEIVGEIVNI
>DmelCSPA29a
MWHCELKVFLILWTVSQVYIPCWGKKFISRFESINGIEGEKETLFTCSVRLVGRERMLNGSIMHQVDLD
DSFDVWMDILHFKNGEWAQGNIKVRTKPCDWFTNYFGKYFLPLVKDSNLPPIQEMCVFPKGEYYLRI

TKIEPQNWPPILYRGLNQFNINYVRDGKSTGGIQFVIDLEDSTL
>DmelCSPB38b
MIRGLVILVLALANTWATDYNALIDDEGIYVKCSEAPAGTLGPRDVFNIDNMVMHMEPEGIYVSGNM
TVKLNFLPSDRISARFSVMHYERGSWQPTMFNLHSPNFCEVMFDEDQYWFKYWFRYIRNKEEIREKC
LKVKDTVLVYDEFLMVLHLENVNTSNLQGRYKAVITLEAFDEHNVRRPSSLCVEIRGDLERVT
>DmelCSPB38a
MISISTILALVVSSVWATDYTLEFEDSDLYSECSEKLPGAIGLREAFDMRNIVTELDIDGLHLSGNCTTIW
DVPSTDRISLRMTVMHFDRGTWQPTVFNTYARDFCAVMFDKELSWYKYWLKYFANREEISEKCIGTK
GTVLVYKPFIVKPLIQNVIGPIYRGRVKAIFNFESFDKNNVKGATDVCFEVRGQVEKIK
>DmelCSPB42b
MIFVLLLLLGVTSSWATDYELLLEDPDIFSTCTDGPPGSINIRQALNLDDIVIDQKGDILHVSGNATVVW
DVQPTDRITARLDVFHFNRGTWEPTVFSMATQNFCSIMYDKNQYWYKYWTRFITNRHEVEKKCFRG
PDTVLVHEPFDLILKFENFRGPLLRGRHKLVILFNALDERNIPRPNPICLEIIGEPLKLQ
>DmelCSPB42c
MRTLMLFVFGFASSWAADYELLLEDPDIFSPCTEPPPGSIGFHDAFDIGDLVVDQDMDIIHLSESVTSI
WDVEPTDRISARFAIMHYNRGSWEPTVFSMATPDFCASMFDENQSWFKYWTKHISNRDEVMEKCF
KTRGTVIMHNPFDLQLRLTDIRGATLRGRYKAVVTFEAVDEKDVPRRNSICFEIRGEAEKIN
>DmelCSPB53a
MLELILILNVVHLSLQISYEFEIEDESIYSDCSDVPPGTLNISGLFDLTNYTTTITADGLSVSGNMTSVFNA
QPTDRIELTGNLLFFDRGAWQPTTLNMMVRDFCAVMYDKKQLWYTDWSSHVVNRDEIKDNCIKVP
GTLFLIESYNMKLVFGSGIPLGTGRYSIRVQVFAYDQKGKKRPNNVCYEVKGNFYKESNICISKPILITIN
VLAESLKSQMSSTNHANI
>DmelCSPB53b
MSQQLFRIWQLLVLMAFIPHSIEVSYEFTMEDERITSDCQNEPPKTLNIDGLFDMSNIDFEMAEDGVQ
LSGYKTVVWDIQPTDRVELQGSVQYFDRGTWQPTTLNMLVKDFCHVLFDKKQVWYDAYSKHITNS
AQINNTCFRVKGSVIEFETYTIGLEFGSGIPLHRGRYAIRLKFRAFDKNGKVRPNEICFEIKGQFSKKSLG
>DmelCSPA84a
MKQFLFCVLIMLIGKTCALIPRTYETRFISITSNGTNLFDFSQIRFLGRERMANGTFELKEDLDNESFSVV
GETFIDSVGDGEYKQLPFTAPKQSVCTALKAYWSYFEPSIKYGVKTDFPAHTHPCPLPKGIYYIKDVVLK
NDNWPVIMPRGYLKAVANLFKNDEYGGSLEIVSQISDLS
>DmelCSPA86a
MLKYTVLVLLIGIPKLLLQAQMSYEAIFVSVTSEENSKPFDLSNLRLIGRERILNGTFEILEDLDDEHFQISV
EIYTNPARDGNYKLLPMSVPRQGVCTFFKKYGFYFRDCIKNGINTDLFLNTTSCLFPKGHYYLKNVTIN
VQNWPKIMQRGLCRHIAFFYKNNVPMGSYNLTSSIEDRAPNFNLRPL
>DmelCSPA56a
MSFWWMIQSLLILWTVSVGSKKSNYEVRFESIDAVKGSTETLFLYQLRLLGRNRMINGTLIFLEDLDETF
DVLFESHAFKNGYWVKGIVNAAASKPCEFFNRYYISFFLVKSTESNLPTTGAEMCPFRKGTYFVKNGV
VSTEDWPPIVFKGLNRFTISYLKNGECVGGVQLTISIAEIIT
>DmelCSPA46a
MSLELHKVLLVLSIAHSALVRAGLECRIESISKVFGDNETLFEFNFRVIGRQRLLNGTLNFHVDLDDDYE
MSNEVLALKDGEWESTSVSARFKTCKYMAVIYDKYFAVSFKDSNIPKGTEACPIKKGEYYARNVEVIAD
NWAHYAKLGLVRSNMLVRKNNVVYGGFDIVLVLSQKIV
>DmelCSPB74a
MDFQFSVLCQILIFLTAAQPSIGGYFEYVLDDESVFSECFNTPPGYANVSGLFDVSTVNFEMGPEGVHI

DGHVTSTWDIQPTDRIEGRLNVVHMDRGTWQPTVLNMVCKDFCKTFLDPNQYWYNVFPKHIINKD
EARQKCLNYKGTVYFVEPYTLQMHFGLGLTLPSGRNRMVINLVAIDENNVTRPNGICFEVKGDFFKIE
>LmigCSP1
KYDNIDLDDILHNDRLLKKYHECLVSSSDASCTPDGKELKAVIPDALTNECAKCNEKQKAGAEKVIKFL
VKEKPDLWEPLEKKYDPSGSFRQKYGPELKKVSA
>LmigCSP2
MQALTLVLFALVASAAAYTTKYDNIDLDEILNNERLLKKYHECLMSDSDASCTPDGKELKVSIPDALVT
DCSKCNEKQKEGSNKVIRFLIQKKEDLWKPLQAKYDPEGTYLKKHPELLSA
>LmigCSP3
MKSCALALLLVGLVAAAAAYTTKYDNIDLDEILHNDRLLNKYHECLLSDTDTPCTADGKELKAAIPDAL
TNECAKCNEKQKNGAEKVIRFLIKEKPDLWTPLEKKYDPNGTYRQKYGEELKKVSS
>LmigCSP4
MQKCTLALLLACLVAATAAYTTKYDNIDLDDVLHNDRLLKKYHECLLSDSDASCTPDGKELKAAIPDA
LTNECAQCNEKQKAGAEKVIRFLIKEKPDLWEPLEKKYDPTGSFRQKYDQELKRVSA
>LmigCSP5
MQKCTLALLLACLVAAAAAYTTKYDNIDLDDVLHNDRLLKKYHECLLSDSDASCTPDGKELKAAIPDA
LTNECAQCNEKQKAGAEKVIRFLIKEKPDLWTPLEKKYDPTGSFRQKYDQELKRVSA
>SexiCSP1
MRAVLFLCALVHVVVGQDVNDMVNMPKYDQRYDYLDVDAIFANKRLVRNYVDCLINAVRCTPEGK
ALKRILPEALRTKCVRCTERQKRTAVKVIKRLKNEYPDEWSKLASRWDPTGDFTRYFEEFLAKEHYNTIP
GSGSALPTSAPIAPPRVSPLPPSTTPTPGPTESTPLRPLVLNRFGDDGELMMGSPSSAGVTPRPMTQA
TTRPSTTTKTPSTRPVPPRPTMMTWAGAASNTQSTRFPLRPVSEISPPYSTAITLIDQIGYKIIKTTELVTD
ILRNTVRAVVGR
>SexiCSP2
MQYIFRYGRDEGVTAQRRVERSSREIRYKMKLIIVVALCLVAVAWAKPASTYTDKWDNINVDEILESQ
RLLKAYVDCLLDRGRCTPDGKALKETLPDALEHECSKCTEKQKKSSDKVIRHLVNKRPDLWQELSGKY
DPENIYQERYKNQLDAVKRQ
>SexiCSP3
MMKYIALAMMLLVYLTIQSNAVETSTYTTKYDGIDLDEILNNERLLTGYVNCLMDNGPCTADGKELKR
NLPDAIENDCKKCTDRQREGADRVMHYLIDHRPEDWTKLEKKYNSDGSYKMKYLSRKPAEDSKETNT
TKSEEDTKNETKEFT
>SexiCSP4
MMQKSFITMILLMYLTIQSNATETSTYTTKYDGIDLDEILSNDRLLTGYVNCLMDIGPCTADGKELKKN
LPDAIENDCKKCTERQREGADRVCHYLIDNKPEDWTKLEEKYKSDGSYRAKYLASKQTKDEKESNATK
SSEDTNNVSKE
>SexiCSP5
MKNMIALFVWILFHSVLSEEPNKYTSMYDNIDLQEVVHNERLLKNYVNCLLEEERCTPDGAELRRNLP
DAVENDCKKCTQKQKEGADFMMQYLIDNKPEYWNLLQKKYDPSGSYRTKYLEAKNGAVQEENAKE
SSEHHII
>SexiCSP7
MKYIAVVVLFAIVGLAYCRPDDGQYTTKYDNVDIDQILKSDRLINSYFKCLMTGDGCTPDGAELKKILP
DAIETGCSKCSRKHKDGSKKIINHIIINKPDLWKQLEAKFDPQGTYKQKYKAELEKEGFKF
>SexiCSP8
MKFVILLGLLVVYFWAFTEAEEYKSQYDDLDVDEILRSKRLLKNYVDCLLDKGRCPPPAQELKEHLDDA

LKTECKKCSEKQKKVGTKVINFMIENHPDMFHLLEARYDPEGVYMAKWKEMKKNTDETKA
>SexiCSP9
MKSFIVLCLFGLAAVAMARPDGSTYTDRYDNINLDEILGNRRLLTPYIKCILEEGKCTPDGKELKSHIREA
LEQNCAKCTDAQRNGTRRVLGHLINNEEESWNRLKAKYDPQSKYTVKYELELRKLKQ
>SexiCSP11
MKFVLVLCLMAAAVLADDEKYTSKYDNIDLDEILTNKRLLTAYVNCIMERGKCSPEGKELKEHLVDAIE
TGCTKCTENQEKGAYKVIEHLIKNELDIWRELTGKYDPSGKWRKTYEDRAKANGIIIPE
>SexiCSP12
MKSMIVLCVLSVAALVVARPDDSHYTDRYDNVDLDEILSNRRLLVPYIKCILDQGKCAPDAKELKEHIR
EALENECGKCTETQKKGTRRVIEYLINNEEEYWNELTAKYDPERKYTAKYEKELKKIKA
>SexiCSP13
MKVLLVLCILAAAALADDKYTDKYDNINLDEILENKRLLLAYVNCVMERGKCSPEGKELKEHLQDAIET
GCTKCTEAQEKGAYKVIEHLIKNELDIWRELAAKYDPKGDWRKKYEDRARANGIQIPE
>SexiCSP14
MKVVFLVCVLAAVVYSHPHDSHYTDKYDNIDLDEILNNKKILTSYINCCLDLGKCTPDGKELKSHIREAL
ENKCGKCTEAQKNGTRKVMTHLINFEPDYWNQLCAKYDPEGKYKAMYEKEYKTLVH
>SexiCSP15
MKLIVAVALLCLVAESWAASTYTDKWDNINVDEILESQRLLKAYVDCLLDRGRCTPDGKALKETLPDA
LENECSKCTDKQKSGSDKVIRHLVNKRPEMWKELSAKYDPNNIYQDRYKDKIEAVKGQ
>SexiCSP18
MMKANCFLIVTLIAVVAADFYNSKYDSFDVQPLLENDRILLSYTKCFLDQGPCTPDAKDFKKVIPEALE
TTCGKCSPKQKQLIKTVIKAVISRHPDAWDQLTEKYDTDKKYKESFDKFLAEQD
>SexiCSP19
MNFLVLSIVVTMAAFVAAETYTDRYDHINIDEIIENRKLLVPYIKCTLDQGRCTPEGRELKAHIKDAMQT
SCSKCTEKQKKGARKVVKHIRAKEQEYWKQILAKYDPEDQYKENYETFLAAED
>SexiCSP20
MKCIYVLSVLLAFAAVQAEDKYSTENDDLDIEAVVADLDTLKGFVGCFMDAMTCHAVAADFKKDIPD
AVATSCAKCTNAQKHIFHKFLLGLKEKLPSDYEAFKKKFDPQGQYFEALEAAVASS
>SexiCSP21
MRVLVVLSCLVVVAFAADKYNPKYDNFDVDTLISNDRLLKAYINCFLEKGRCTPEGSDFKKALPEAIETT
CAKCTDKQKGNIRKVIKAIQQKHPKEWEDLVKKNDPSGKHRGNFDKFIQGSS
>SexiCSP22
MNALLIAVFALAAPLVLGYDEKYDKLDVDKILGDDALFTAYIDCMLDKGPCSVEHSADFRQLLPEVIST
ACAKCSAIQRQNVRKTVKALSEKKPDDFAQFRTKFDPKGEYEKAFSAFVIGTD
>SexiCSP23
MRSWLLCLCVLTVVVSCYSQANRYENFNPDAIVQNDRILLAYYKCVMDKGPCTRDGKNFKRVLPETL
ATACGRCNPKQKTIVRKLLLGIRSKSEPRFLELLDKYNPDRSNRDALYAFLVTGA
>SexiCSP24
MKVVLLTLCFALGVLAQDQYESANDNFDISEVIGNDRLLHAYANCLLNKGPCTPEVKQVKEKLPEALE
TRCAKCTDKQKQMGKALAQEVKKNHPDIWKQLVAMYDPQGKYQQAWKDFLQE
>SexiCSP25
MKVALLTLCFALGVLAQDTYENANDNFDISEVLNNERLLNSYAKCLLNKGPCTPEVKQVKEKLPEALE
TRCAKCTDKQKQMGKALAQEVKKNHPDIWQQLVAMYDPEGKYQQAWKDFLQE
>SexiCSP28

MKVFVVLSALIAFTAAASLTPAELDLAEAFDYDALFSNDEQRKLVFDCILGKGDCGDYQKVAEISRKILE
SQCADCNASQKAKYETVLKTIQTKYEPFYNELLKNVAAKKE
>SexiCSP29
MQIKYALVLCCVAAVSVAQSQRPPVSDTALDDALQDKRFIQRQLKCALGEGPCDPIGKRLKTLAPLVL
RGACPQCTPQETKQIQRTLSYVQRNYPQQWAKIVRQYAG
>SexiCSP30
MQVVVVLVVACVGLVAGLHVQAGPQMTDAQLEQTLADKSTMQRHIKCALGEGPCDPVGRRLRTL
APLVLRGACPQCSMQETRQIRRTLAFVQRNYPWEWAKIVRQYG
>FoccCSP1
MVRAGPLACLALSVLVLAEAVDKYEEGRFAHINVDEVLGNQRILGSFIKCFLDEGPCTADSRDMKKLL
PEVIDSKCESCTDNQKKMMAKAVKHVKDNRPDDWERLTNKYDPDHTKTEDIDKFMSDALAM*
>FintCSP1
MVRAGPLACLALSVLVLAEAVDKYEEGRFAHINVDEVLGNQRILGSFIKCFLDEGPCTADSRDMKKLL
PEVIDSKCESCTDNQKKMMAKAVKHVKDNRPEDWERLTNKYDPDHAKTEDIDKFMSDALAM*
>FoccCSP2
MAKMLLCLAVVALAALATAAPKPDEKFTTKYDNINIDEILANKRLLNNYLNCILDKPKARCTADALELK
KSIPDALTNECAKCSEKQKELSEKVVRHLVDHEKTSWEEVKAKFDPTGIYEKRYEKIAQEKGVAV*
>FintCSP2
MAKMLLCLAVMALAALATAAPKPDEKFTTKYDNINIDEILANKRLLNNYLNCILDKPKARCTADALELK
KSIPDALTNECAKCSEKQKELSEKVGRHLVDHEKTSWEEVKAKFDPTGIYEKRYEKIAQEKGVAV*
>FoccCSP3
MSRRGLFLAAAPLATVLALAAAKPQPGKLDSVDVDAVLKNKRLFNNYVKCIMDQAPCSAEGSELKES
LPKDLATKCASCSDTEKSRAKKVINYLKKERKEVWEDFKKKYDPESKWGDLEWASSSW
>FintCSP3
MSRTGLLLAAAALAAVLALAAAKPQPGKLDSVDVDTVLKNKRLFDNYVKCIMDKAPCSAEGSELKESL
PKDLSTKCAACTDSEKARAKKVINHIKKERKDVWEDFKKKYDPEGKWGDLDWVSAA*
>FoccCSP4
MKCATVVVLAAALCCAAVLASGPAAFEGVDVAALLKNESAVAGYVKCILGTGECNESAKRLQAIVPN
ALQGKCSQCSEQQKVIIGTIVNTMQSKFPNEWEKLLQKYDPEKKYRADILALAKAVPTGAPTGTAATT
AKPAKHW
>FintCSP4
MKCATVVVLAATLCCAAVLASGPAAFEGVDVAALLKNESAVAGYVKCILGTGECNESAKRLQAIVPN
ALQGKCSQCSEQQKVIIGTIVNTMQSKFPNEWEKLLQKYDPEKKYRADILALAKAVPTGAPTGTAATT
DKPAKHW
>FoccCSP5
MKSVPTVCLCLCLAALAALALALADDKYTNKFDNVNVDDILKNQSPLDNYFKCLMDKGRCTPDGAELKKS
LPDALTSRCSKCTEKQKMQTEKVVSFLIEKKPVLWKELKAKYDPEGSYEATYKQDAVSHGIKV*
>FintCSP5
MKSAACVCLCLCLAALAALALALAEDKYTNKFDNVNVDDILKNQRLLDNYFKCLMDKGRCTPDGAELKKS
LPDALTSRCSKCTEKQKMQTEKVVRFLIEKKPVLWQELKAKYDPQGQYEATYKQEAVAHGIKV*
>FoccCSP6
MNSVSAPALLLLVVVALATVHAAPSDGPASTECPAGIKAAKAGPAAQGVKYTTKYDNINVDMVLHN
ERLLNNYMECLMDRSACSKEGQLLKEIIPDALQTDCARCSEKQKQIAGTIMSYLLQYKKKYWDELLCK
YDPEGNFRKKYEVDEDDEDDEE*

>FintCSP6
MKSTAVLLLVALAASAVLAAPKDATKDAKKGTTQCPPGIKPAKAGPAAQGGTYTTKYDNLNVDMVL
HNERLLKNYIDCLMDRKPCSKEGQLLKEIIPDALQTECSRCSEKQKQIAGTIMSYLLQYKKNYWDELLC
KYDPEGTFREKYEVDEDDEEED
>FoccCSP7
MVPLRTAVAVLVLAAVARAALVQRVRRDDDDDEKYTSSFDNVDLDTVLNSDRLLTNYFRCIMDEGP
CTPDAKELKRVIPEALSNKCAKCSERHRAGAEKVLTFLIKNREAEWTRLEKKYDPSGQYRKLYEAEAAQ
RGLKI*
>FoccCSP8
MTASTMSLVAIAAVAAVVVLAASPSAAAPQSMANFGKGNINAYLMDPMLVSKTISCVEGVGPCTRL
GNSLKSAIPTVLNNCKGCDQQQAANASKLIDYIQSNYPRDYDMILAKYRNAPPRG*
>TcasCSP1
MLILQIAHLCAQFCLLAAIFTCVKPQLTRISDEAIESTLNDRRYLLRQLKCATGEAPCDPVGRRLKSLAP
LVLRGSCPQCTPQEMKQIQKVLAFVQKNYPKEWNKILHQYAG

>TcasCSP2
MKIIILAVLIATAVAATYDVYPTKYDNVDIDAILHNKRLFDNYLQCLLKKGKCNEEAAILRDVIPDALIT
GCRKCNDHQKVSVEKVIRFLIKERNSDWQQLISVYDPKGEYQTQYAHYLEKI
>TcasCSP3
MLFTVFLVLTCAHVVFLEEYVIPDNIDIDDILSNERLLKNYVNCLLDKGRCTPEGKKLKSTIPEALSTDC
AKCNEKVKANVRKVLHHLIDNKPDMWKQLEAKYDPSGEYRSKYKDELEKNGIHV

>TcasCSP4
MYSYLIPLYLFLFVHYGWSEDTTHKYTTKYDNIDLENVVKNERLLKSYVDCLLEKGRCSPDGLELKKNM
P
DAIETDCSKCSEKQKEGSDFIMRYLIDNKPDYWKALEAKYDPDGTYKKRYFESQKDEVSKVEA

>TcasCSP5
MKTFVILFFGVFFIIFSDFVNGKTLHRSTRDDKYTTRYDNVDVDRILHSKRLLLNYINCLLEKGPCSPEG
RELKKILPDALVTNCSKCSEVQKKQAGKILTFVLLNYRNEWNQLVAKYDPDGIYRKQYEIDDDYDYSEL
D
SAKK

>TcasCSP6
MIPLIAIAGILAVSAAPAEFYESRYDHLDVESILNNRRMVNYYAACLLSKGPCPPQGVDLKRVLPEALQ
T
NCAKCTEKQRTAAYRSIKRLKKEYPKIWEQLRAVWDPDDVFIRKFETSFESGKPSGVISTNTSPPSPILS
NRFGENEEADAASNVISSTPLPPTTSTTTRTTLTTKFTTKPSTKPTNKPVVVTKPPQAPPFATVGANLQ
A
TVSFGTNLVGGIVRSLGTLGSRVVESGTKLANMVISAAIRP

>TcasCSP7
MKLISAVILCAFLVAVSAAENKYTNKYDNVDVDKILNNDRVLTNYIKCLMDEGPCTSEGRELKKTLPDA
L

SSGCTKCNQKQKETAEKVIRHLTQKRARDWERLSKKYDPQGQYKKRYEEHVATSRAA

>TcasCSP8
MPLVKSLVVVVLLIGVVYQVQGQLGLAGNNYIEKQLLCALDKAPCDALGNQIKGALPEIIGKNCERCD
SR
QVANARRIARYVQTKHPDVWNALVKKYSV

>TcasCSP9
MTAIVFLLALACLKTYVSSQEYLVPQNIDVDEILKNDRLTRNYLDCVLGKGKCTPEGEELKKDIPEALQN
GCAKCNEKHKEGVRKVIHHLIENKPNWWQELESKFDPQGEYKKKYDELLKKEGLAN

>TcasCSP10
MKTFVLVAFAAVLGLALARPQEKYTTKYDNIDLEEILKSDRLLKNYFNCLMERGTCSPDGEELKKALPD
A
LHSGCSKCTEKQKEGSRKIIHYLIDNKRDWWNELEAKYDKDGVYRQKYKDVIEKEGIKL

>TcasCSP11
MKTLVPLLFFVIAIASSLAENSKYTTKYDNVDLDEIIKSDRLLKNYVNCLLEKGKCTPDGAELKRHLPDA
LHTECSKCSETQKNGSKKIMRHLIDHKRDWWNELEEKYDKEGEYRKKYEAEIKGKKD
>TcasCSP12
MKTLVLVLFVAVLSVVFAADKYTTKYDNIDLNQILKSDRLLKNYVNCLLDRGKCSPDGQELKNNLADA
LQ
TSCSKCSQRQKDGSRTIIRYLIKNKRDWWNELEAKYDPTGIYKNKYADELKAEGIVL
>TcasCSP13
MFLAIVLVVCACTNVLSEEYTNQYNDELDAALKSERLMKSYFECLLGTGKCTPSGEELKKDIPDALKNE
C
AKCNDKHKEGIRKVIHYLVKQKPEWWEQLQKKFDPQGIYKKRYQNYLDKEGLKA

>TcasCSP14
MFATSALFAFICIQGLVSAEEYLVPQNIDLDEILKNDRLTRNYIDCILGKGKCTPEGEELKRDIPEALQN
ECAKCNEKHKEGVRKVLHHLIKNKPNWWQELEAKFDPKGEYKQKYNKLLEKEGLQA

>TcasCSP15
MIFKIHFLVFGALLTYVSSVEYLILREIDTILKNDQMTRNYLDCVLDKGKCTKEAEKLKKGITETMKNGC
VKCEQKQKEDVHKVFQHLMIHRPNWWHELETKFNPHHEIKLQHLHQSKFNPHEEVKLQHLHQFPH
HDFLE
REGFIR

>TcasCSP16
MTAIVFLLALACLKTYVSSQEYLVPQNIDVDEILKNDRLTRNYLDCVLGKGKCTPEGEELKKDIPEALQN
GCAKCNEKHKEGVRKVIHHLIENKPNWWQELESKFDPQGEYKKKYDELLKKEGLAN
>TcasCSP17
MFKVLFVVFACVQAYVYAEEYTVPQNIDIDEILKNDRLTKNYLDCILEKGKCTPEGEELKKDIPDALQNE
CAKCNEKHKEGVRKVIRHLIKNKPSWWQELQEKYDPKGEYKSRYNHFLEEEGLN

>TcasCSP18
MLFTVFLVLTCAHVVFLEEYVIPDNIDIDDILSNERLLKNYVNCLLDKGRCTPEGKKLKSTIPEALSTDC
AKCNEKVKANVRKVLHHLIDNKPDMWKQLEAKYDPSGEYRSKYKDELEKNGIHV

>TcasCSP19
MKFFIAFLMLLGAVWCEQYTTKYDNINVDEILASERLLKNYFNCIMDRGACTPDADELKRVLPDALKS
DC
AKCSEKQKEMTKKVIHFLSHNKQQMWKELTAKYDPDGIYFEKYKDKFDS

>TcasCSP20
MRFFVIFFVACVSVALARPEDQYTIKYDNVNLKEILQSDRLTENYVNCLLEKKPCTPDGEELKRVLPDAL
KTSCAKCTDKQKQGAKTVIQHLYKNKQDWWKQLEAKYDPEHTYVKAHEDELKAL
