# Supplementary material for: Comparison and Functional Analysis of Odorant-Binding Proteins and Chemosensory Proteins in Two Closely Related Thrips Species, Frankliniella occidentalis and Frankliniella intonsa (Thysanoptera: Thripidae) Based on Antennal Transcriptome Analysis
Source: Int J Mol Sci. 2022 Nov 11;23(22):13900. doi: 10.3390/ijms232213900 (PMC9692942; doi:10.3390/ijms232213900)
Supplement: Supplementary file 1 [file ijms-23-13900-s001.zip › supplementary tables.pdf]

Table S1 Information for nine samples used for transcriptome analysis

Sample	Clean read number	Base number	GC content (%)	%≥Q30
FTF1	39,000,448	5.37G	34.41	91.53
FTF2	49,432,170	7.1 G	48.33	94.02
FTF3	51,572,006	6.89 G	31.73	88.99
WFTF1	66,120,138	9.68 G	51.57	94.84
WFTF2	70,084,582	10.27 G	50.58	95.19
WFTF3	66,363,978	9.74 G	51.10	95.32

Table S2 An overview of the transcriptome sequencing and assembly of *F. occidentalis* and *F. intonsa* antennae transcriptome.

	Total sequence number	GC%	Min length	Median length	Max Length	Total assembled Bases	N50
Transcript	74,380	45.31	201	514	10408	59,876,343	1,257
Gene	48,889	44.56	201	458	10408	36,818,905	1,188

Table S3 Annotation information for *F. occidentalis* and *F. intonsa* unigenes in different databases.

Database	Num	Ratio (%)
All	48,889	100.00
GO	18,484	37.81
KEGG	11,111	22.73
Pfam	18,772	38.40
swissprot	16,468	33.68
eggNOG	22,490	46.00
NR	21,362	43.69

Table S4 Pocket parameters of OBPs and CSPs in in *F. occidentalis* and *F. intonsa*.

Species	Pocket name	Volume	Surface	pocket atoms	Hydrophobicity ratio	Simple Score
Frankliniella occidentalis	FoccOBP1	599.23	866.79	120	0.54	0.45
	FoccOBP2	703.68	1,085.93	171	0.44	0.48
	FoccOBP3	1,350.46	1,607.61	266	0.44	0.66
	FoccOBP4	1,409.41	1,792.74	274	0.44	0.66
	FoccOBP5	1,039.1	1,345.69	168	0.48	0.68
	FoccOBP6	934.27	1,155.44	221	0.39	0.61
	FoccOBP7	768.96	1,375.26	175	0.53	0.56
	FoccCSP1	1,326.02	1,665.12	247	0.38	0.64
	FoccCSP2	1,379.39	1,453.56	265	0.46	0.67
	FoccCSP3	1,211.46	1,468.77	240	0.51	0.69
	FoccCSP4	558.72	881.04	172	0.46	0.39
	FoccCSP5	1,219.78	1,391.13	228	0.44	0.66
	FoccCSP6	1,219.78	1,391.13	228	0.44	0.66
	FoccCSP7	1,597.12	1,921.07	296	0.45	0.67
	FoccCSP8	788.61	1,429.47	212	0.37	0.51
Frankliniella intonsa	FintOBP1	634.75	779.3	142	0.48	0.45
	FintOBP2	929.66	1,316.53	197	0.48	0.64
	FintOBP3	1,232.96	1,642.84	251	0.48	0.68
	FintOBP4	1,356.42	1,991.59	311	0.44	0.66
	FintOBP5	1,067.65	1,420.72	165	0.41	0.65
	FintOBP6	1,203.07	1,329.34	258	0.39	0.64
	FintCSP1	1,352.64	1,630.22	253	0.37	0.63
	FintCSP2	1,439.55	1,525.58	265	0.46	0.67
	FintCSP3	1,482.04	1,950.44	280	0.46	0.67
	FintCSP4	558.72	881.04	172	0.46	0.39
	FintCSP5	1,235.14	1,384.74	233	0.42	0.66
	FintCSP6	1,055.17	1,002.28	199	0.45	0.67

Table S5 Model templates of OBP and CSP genes of *F. occidentalis* and *F. intonsa*.

Species	Gene name	Model template	Sequence identify	GMQE
Frankliniella occidentalis	FoccOBP1	6jpm.1.A	30.36%	0.39
	FoccOBP2	2wck.1.A	21.24%	0.25
	FoccOBP3	1dqe.1.A	18.42%	0.35
	FoccOBP4	3bfb.1.A	29.06%	0.61
	FoccOBP5	6qq4.2.A	30.51%	0.56
	FoccOBP6	4f7f.4.A	18.92%	0.46
	FoccOBP7	4z45.1.A	20.99%	0.19
	FoccCSP1	1n8v.2.A	31.07%	0.50
	FoccCSP2	1n8v.2.A	46.79%	0.49
	FoccCSP3	1n8v.2.A	35.71%	0.52
	FoccCSP4	1n8v.2.A	22.73%	0.45
	FoccCSP5	1n8v.2.A	44.04%	0.52
	FoccCSP6	1n8v.2.A	45.71%	0.42
	FoccCSP7	1n8v.2.A	44.44%	0.48
	FoccCSP8	2gvs.1.A	30.26%	0.41
Frankliniella intonsa	FintOBP1	6jpm.1.A	24.58%	0.49
	FintOBP2	2wck.1.A	18.75%	0.22
	FintOBP3	6qq4.2.A	18.26%	0.42
	FintOBP4	3bfb.1.A	28.21%	0.62
	FintOBP5	6qq4.2.A	30.51%	0.61
	FintOBP6	4f7f.3.B	19.64%	0.46
	FintCSP1	1n8v.2.A	32.04%	0.50
	FintCSP2	1n8v.2.A	46.79%	0.49
	FintCSP3	1n8v.2.A	37.50%	0.52
	FintCSP4	1n8v.2.A	22.73%	0.46
	FintCSP5	1n8v.2.A	47.71%	0.52
	FintCSP6	1n8v.2.A	41.90%	0.41

Table S6 Primer sequences used for RT-qPCR in *F. occidentalis* and *F. intonsa*.

Species	Gene name	Forward Primer (5'~3')	Reverse Primer (5'~3')
Frankliniella occidentalis	FoccOBP1	TCATCAACGCCTTCACTGAG	GGTCGGGTCAACTTTCTTGA
	FoccOBP2	CCCAACATGAATTTCCCTGA	AGTCCAGGTATGCTCGGTTG
	FoccOBP3	AAGGAGCTGATGCCTCGATA	CGTTGCCCTCTTTCATCATT
	FoccOBP4	CGATATGTTGCTCACCATGC	TCTGGGGCCTTCTCATACAG
	FoccOBP5	AAAGCGAGACGAAGCTCAAG	CGCTTTTCGGTACTCTTTCG
	FoccOBP6	AAGAAGCGCAAGCTCATGTC	TCCCCTTGTACGAGTCCTTG
	FoccOBP7	CTTCACCGAGGACGAGAGAC	GCGTAAAGCTTGACCAGTCC
	FoccCSP1	TGCCAGAAGTGATTGACAGC	CCAGTCGTCTGGACGGTTAT
	FoccCSP2	AAGCGCCTCCTCAACAACTA	CTTCTGCTTCTCGGAGCACT
	FoccCSP3	AGGAGAGGCTGCCTAAGGAC	TCTTGAAGTCCTCCCACACC
	FoccCSP4	GGCTACGTCAAGTGCATCCT	GATGATCACCTTCTGCTGCTC
	FoccCSP5	CACCAACAAGTTCGACAACG	GGCAGCGACTTTTTGAGTTC
	FoccCSP6	TACATGGAATGCCTGATGGA	GCGATCTGCTTCTGCTTCTC
	FoccCSP7	TACACGTCGAGGTTCGACAA	TGATGCAGCGGAAGTAGTTG
	FoccCSP8	ACAGCCTGAAGAGTGCCATC	GGCGAGGATCATGTCGTAGT
Frankliniella intonsa	FintOBP1	CCTCTTCATCAACTGCGTCA	CCATGTAAAAAGGCGTGGTT
	FintOBP2	GCAGCAAATTGGTTTGGATT	CGTGTAATTCCTCCCAAACG
	FintOBP3	CCCAGGCAGACAAGAAAAAG	TGTCGTTCCCTTCCTTCATC
	FintOBP4	ATGACTGTGATGGACGACGA	AAATCCTTGCAAGCGTTGAG
	FintOBP5	AAAAAGGAGTGCAAGGAGCA	CGCAGGCCATCAGTTTCTTA
	FintOBP6	GACAAGCTCATCCCGGAGTA	GTTCACCCCTTCCTTCATCA
	FintCSP1	TGCCAGAAGTGATTGACAGC	TAACATGCTTCACGGCTTTG
	FintCSP2	AAGCGCCTCCTCAACAACTA	CTTCTGCTTCTCGGAGCACT
	FintCSP3	AAGCGCCTCTTCGACAACTA	ACTTGGTGGACAGGTCCTTG
	FintCSP4	GGCTACGTCAAGTGCATCCT	GATGATCACCTTCTGCTGCTC
	FintCSP5	CTTCAAGTGCCTGATGGACA	CCTTCTCGGTCTGCATCTTC
	FintCSP6	AGCGCCTGCTCAAGAACTAC	GTCCGGAATGATCTCCTTGA
Reference	β-actin	GGTATCGTCCTGGACTCTGGTG	GGGAAGGGCGTAACCTTCA
